# FSP1 inhibition enhances olaparib sensitivity in BRCA-proficient ovarian cancer patients via a nonferroptosis mechanism

**DOI:** 10.1038/s41418-024-01263-z

**Published:** 2024-02-19

**Authors:** Huixian Miao, Huangyang Meng, Yashuang Zhang, Tian Chen, Lin Zhang, Wenjun Cheng

**Affiliations:** Department of Gynecology, the First Affiliated Hospital of Nanjing Medical University, Jiangsu Province Hospital, Nanjing, 210029 Jiangsu China

**Keywords:** Cancer genetics, Tumour biomarkers, Tumour-suppressor proteins, Gene expression, Gene regulation

## Abstract

Poly ADP-ribose polymerase inhibitors (PARPis) exhibit promising efficacy in patients with BRCA mutations or homologous repair deficiency (HRD) in ovarian cancer (OC). However, less than 40% of patients have HRD, it is vital to expand the indications for PARPis in BRCA-proficient patients. Ferroptosis suppressor protein 1 (FSP1) is a key protein in a newly identified ferroptosis-protective mechanism that occurs in parallel with the GPX4-mediated pathway and is associated with chemoresistance in several cancers. Herein, FSP1 is reported to be negatively correlated with the prognosis in OC patients. Combination therapy comprising olaparib and iFSP1 (a FSP1 inhibitor) strongly inhibited tumour proliferation in BRCA-proficient OC cell lines, patient-derived organoids (PDOs) and xenograft mouse models. Surprisingly, the synergistic killing effect could not be reversed by ferroptosis inhibitors, indicating that mechanisms other than ferroptosis were responsible for the synergistic lethality. In addition, cotreatment was shown to induce increased γH2A.X foci and to impair nonhomologous end joining (NHEJ) activity to a greater extent than did any single drug. Mass spectrometry and immunoprecipitation analyses revealed that FSP1 interacted with Ku70, a classical component recruited to and occupying the end of double-strand breaks (DSBs) in the NHEJ process. FSP1 inhibition decreased Ku70 PARylation, impaired subsequent DNA-PKcs recruitment to the Ku complex at DSB sites and was rescued by restoring PARylation. These findings unprecedentedly reveal a novel role of FSP1 in DNA damage repair and provide new insights into how to sensitize OC patients to PARPi treatment.

## Introduction

Ovarian cancer (OC) is the most lethal gynaecological cancer worldwide [1]. Due to the lack of effective tools for disease screening, most patients present late-stage disease (stage III and IV) at diagnosis. Currently, the standard care for first-line treatment is debulking surgery combined with taxane- and platinum-based chemotherapy. Although most patients respond well initially, the majority eventually experience recurrence, with a 5-year overall survival of less than 40% [2]. One of the latest breakthroughs in OC treatment is the approved use of poly (ADP-ribose) polymerase inhibitors (PARPis), which are recommended as a single agent maintenance therapy in advanced OC [3, 4]. PARPis are selective inhibitors of PARP enzymes and are designed to inhibit PARP activity [5]. PARP enzymes, which are responsible for PARylation of their target proteins, can increase the formation of double-strand DNA breaks (DSBs) in various ways. The repair of these breaks typically relies on homologous recombination (HR) and nonhomologous end joining (NHEJ). Moreover, HR activity is significantly decreased in cells harbouring BRCA1/2 mutations and HR deficiency (HRD). Hence, PARPis exploit cellular vulnerability by causing synthetic lethality in tumour cells.

Although the introduction of PARPis has led to encouraging progress in determining the prognosis of OC patients, treatment with PARPis has been challenging [6]. On the one hand, the clinical benefit of PARPis is restricted to a limited population of patients since only approximately 30% of patients experience HRD in the real world [7]. Although emerging evidence shows the efficacy of PARPis in patients without HRD, it is undeniable that they are not as effective as they are in patients harbouring HRD. On the other hand, disappointingly, drug resistance has developed during clinical practice, even in patients with BRCA1/2 mutations, which inevitably hampers the potential benefit of PARPis [8]. Thus, exploring the feasibility of PARPis in patients without BRCA mutations and discovering new strategies to reverse PARPi resistance in OC are urgently needed.

Ferroptosis is a newly identified form of regulated cell death characterized by excessive iron-dependent lipid peroxidation and differs from apoptosis and necroptosis in terms of genetics, biochemistry and morphology [9]. The well-known intracellular dominant protective pathway against ferroptosis is regulated by glutathione peroxidase 4 (GPX4). Recently, PARPis were shown to promote ferroptosis by downregulating the GPX4-mediated pathway in a p53-dependent manner in BRCA-proficient OC, revealing the important role of PARPis in regulating ferroptosis [10, 11]. Ferroptosis suppressor protein 1 (FSP1), encoded by the apoptosis-inducing factor mitochondria-associated 2 (AIFM2) gene, is another antiferroptosis player in parallel to GPX4 [12, 13]. FSP1 stabilizes phospholipid hydroperoxides by acting as an oxidoreductase to convert ubiquinone (CoQ) into ubiquinol (CoQH2), thereby protecting cells against ferroptosis and cell death [14, 15]. Several studies have revealed that FSP1-mediated antiferroptosis contributes to resistance to radiotherapy and chemotherapy in KRAS- and KEAP1-mutant lung cancers [16, 17]. Additionally, *Cheu* demonstrated that FSP1 inhibition promotes ferroptosis and is associated with dismal clinicopathological features in hepatocellular carcinoma [18]. However, whether FSP1 can synergize with PARPi treatment in BRCA-proficient OC is unknown. Further research is needed to determine the potential ability of these agents to enhance the therapeutic efficacy of PARP inhibitors in this patient population.

iFSP1 is an FSP1 inhibitor that has been screened from among 30,000 compounds based on its ability to induce FSP1-dependent cell death [12]. However, few studies have explored the role of FSP1 and iFSP1 in OC. In the present study, a high level of FSP1 was reported to be associated with a poor prognosis in OC patients. We unprecedentedly demonstrated the participation of FSP1 in the PARylation of Ku70, which was needed for subsequent DNA-PKcs recruitment and NHEJ repair completion. Additionally, iFSP1 has synergistic killing effects with olaparib through suppressing the PARylation of Ku70 but not via ferroptosis in OC cell lines, patient-derived organoids (PDOs) and xenograft mouse models. These findings provide new insights for sensitizing OC patients to PARPi treatment by targeting FSP1 and improving patient outcomes.

## Material and methods

### Patient samples and establishment of PDOs

The 10 patient-derived organoids (PDO) and their parental tumours were obtained from newly diagnosed OC patients at the First Affiliated Hospital of Nanjing Medical University. Institutional ethics approval was obtained (2020-MD-061.A1). The basic characteristics of the patients are shown in supplementary Table S1. OC PDOs were established according to our previously reported protocol [19]. The patients enroled in this study lacked BRCA mutations, as determined through their sequencing reports. The OC cell lines HO-8910, A2780, SKOV3 and OVCAR3 were all derived from BRCA-proficient patients [20].

### Immunohistochemistry (IHC) and immunofluorescence (IF) staining

Paraffin-embedded tissue sections were obtained from the OC patients and subjected to IHC. The streptavidin-biotin labelling procedure was performed as previously reported [19]. For immunostaining, the tissues were permeabilized with 0.5% Triton X-100 for 30 min at room temperature after a series of tissue antigen recovery procedures. After washing three times with 0.03% Tween-20 in PBS (v/v), the tissues were incubated with primary antibodies overnight at 4 °C, followed by incubation with secondary antibodies for 2 h at room temperature. Finally, DAPI was used to visualize the nuclei. Images were acquired using a Thunder Imaging System (Leica, Germany) and were quantified if necessary. The primary antibodies used are listed in Supplementary Table S2.

### RNA extraction and quantitative reverse transcription-PCR

Total RNA was extracted with a RNeasy kit (Beyotime, China) and reverse transcribed with a cDNA synthesis kit (Vazyme, China) according to the manufacturer’s instructions. RT‒PCR was performed on a PCR system (Roche, Switzerland) with SYBR qPCR Master Mix (Vazyme, China). β-Actin was used as an internal control. The sequences of primers used are provided in Supplementary Table S3.

### Organoid viability assay

Organoids were plated in a 96-well plate at an appropriate density in 100 µl of culture medium. After treatment, the organoids were digested and transferred to a 1.5 mL EP tube. After removing the supernatants, 100 µl of CellTiter-Lumi luminescence solution (Beyotime, China) was added to the precipitate. Luminescence, indicating living organoid viability, was assessed on a multifunctional enzyme-linked immunosorbent assay platform. Moreover, to evaluate both the living and dead organoids, a cyto3D Live-Dead Assay Kit (Well Biosciences, China) was used, and the living and dead cells were stained green and red, respectively. Images were captured using a fluorescence microscope.

### Measurements of intracellular lipid oxidation (LPO)

Pretreated HO-8910 and A2780 cells were incubated with the BODIPY^TM^ 581/591 C11 probe (Invitrogen, USA) for 30 min at 37 °C. LPO fluorescence was analyzed through flow cytometry (BD Bioscience). PDOs were treated with probes for 1 h at 37 °C and fixed with 4% paraformaldehyde for 15 min. After three washes with PBS, the PDOs were stained with DAPI. LPO fluorescence was measured by Thunder Imaging System (Leica, Germany).

### RNA sequencing (RNA-seq)

Total RNA was extracted from organoid samples with a RNeasy kit (Beyotime, China), and quality control was performed using Qubit 4.0. After removing rRNA by oligo(dT), libraries were constructed with the Ultra™ II Directional RNA Library Prep Kit for Illumina (E7765S, NEB, MA). These libraries were subsequently sequenced on the Illumina HiSeq X Ten sequencing platform at Novogene Biotechnology, LLC. The raw sequence data are publicly available at the Genome Sequence Archive at the China National Center for Bioinformation/Beijing Institute of Genomics, Chinese Academy of Sciences (GSA-Human: HRA005138; https://ngdc.cncb.ac.cn/gsa-human) [21, 22].

### BLRR system assay

The BLRR system was used to evaluate the efficacy of the HR and NHEJ pathways simultaneously. The protocol was generated based on a previous study [23]. The plasmids used were purchased from Addgene (Catalogue No: 158958). Three replications were conducted for every treatment.

### Cell transfection

To knockdown FSP1, the small interfering RNA (siRNA) siFSP1 and the corresponding control siRNAs were purchased from Tsingke, China, and were transfected into cells with siTran 2.0 siRNA transfection reagent (Origene, USA). The cells were harvested for subsequent analysis after 48 h. To overexpress FSP1, the plasmid targeting FSP1 was cloned and inserted into the PGMLV-CMV-3*Flag-Zsgreen-Puro vector (Genomeditech, China) and transfected into cells with MegaTran 2.0 plasmid DNA transfection reagent (Origene, USA). Puromycin (5 µg/ml) was added to select FSP1-overexpressing cells after 48 h. The sequences of the siRNAs are listed in Supplementary Table S3.

### Co-immunoprecipitation (Co-IP) and mass spectrometry analysis

After being washed with ice-cold PBS twice, 1 × 10^7^ HEK 293T cells were lysed with IP lysis buffer (Beyotime, China) supplemented with protein inhibitors. The supernatants were incubated with IgG or IP antibody at 4 °C to form an immunoprecipitation complex. Then, protein A/G magnetic beads (40 μL; Invitrogen) were added to the supernatants, and the samples were incubated at 4 °C. The target protein complex was obtained and subjected to subsequent analysis. The mass spectrometry analysis was conducted by Novogene Biotechnology, LLC. The antibodies used for immunoblotting are listed in Supplementary Table S2.

### Immunoblotting

Total protein was extracted from cells with RIPA buffer (Beyotime, China). A protease inhibitor was added to the lysates. Protein concentrations were measured using BCA reagent (Beyotime, China). Western blotting (ACE, China) was performed according to previous methods [19]. The antibodies used were given in Supplementary Table S2.

### Animal experiments

Female BALB/c mice aged 4–6 weeks were purchased from the Nanjing Biomedical Research Institute of Nanjing University. After stable infection with lentivirus expressing firefly luciferase (Hanbio, China), HO-8910 cells were injected into the abdominal cavity of nude mice. Seven days later, the mice were treated according to the following planned protocol: DMSO, olaparib (50 mg/kg/d), or iFSP1 (2 mg/kg/d) and combination therapy (*N* = 3). After 14 days of treatment, d-luciferin potassium salt (3 mg per mouse) was injected. Then, the mice were assessed by in vivo fluorescence imaging to evaluate the tumour volume. Finally, the mice were sacrificed, and the tumours were fixed with 4% paraformaldehyde or frozen for further analysis.

### Statistical analysis

Statistical analysis was conducted with GraphPad Prism (version 9.0.2). At least three repetitions were performed for each experiment. The quantification of immunoblot bands and fluorescence were performed with ImageJ. Unpaired or paired two-tailed Student’s t tests were used to calculate the differences between groups. All values were considered significant at a *p* value < 0.05 (*P* < 0.05: *P < 0.01: **P < 0.001: ***).

## Results

### FSP1 overexpression is significantly associated with worse prognosis in OC

**Fig. 1:**
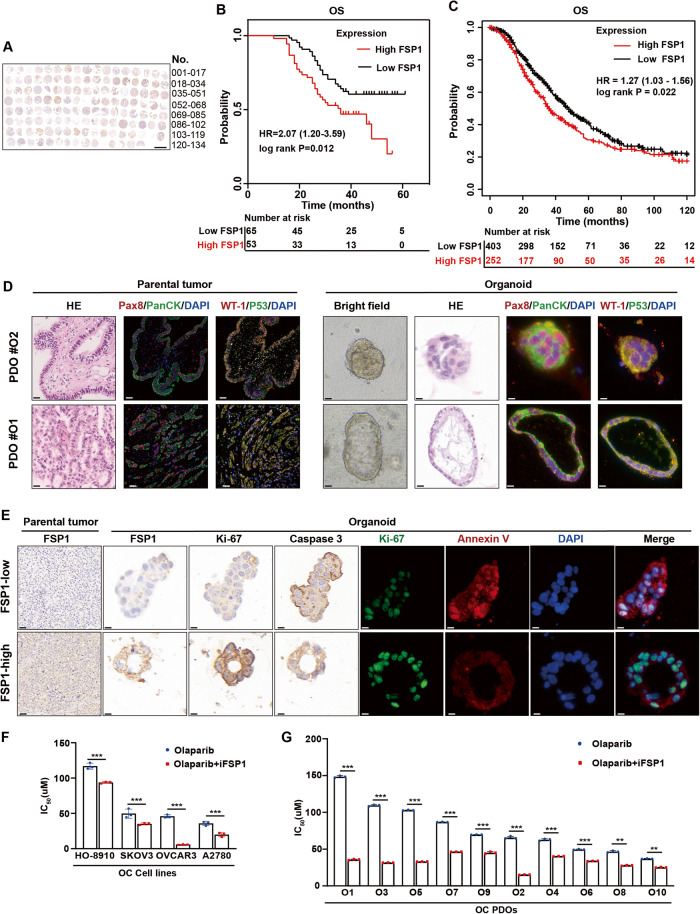
FSP1 overexpression is a risk predictor for poor prognosis in OC and FSP1 inhibitors enhance PARPi sensitivity in BRCA-proficient OC. **A** Immunohistochemistry analysis of FSP1 levels in the tissue microarray of ovary tumours (*N* = 131) and normal ovary (*N* = 3). Patients were grouped into FSP1-high (*N* = 53) or FSP1-low group (*N* = 65) depending on the median expression of FSP1. Scale bar: 2.5 mm. **B** Survival analysis of overall survival (OS) was performed in the FSP1-high/low group (**A**). **C** Survival analysis was performed in the OC patients from the database (https://kmplot.com/analysis/) based on the FSP1 expression. **D** Representative images of H&E staining and immunofluorescence staining of tumour markers (Pax8, PanCK, WT-1 and P53) of paraffin sections from OC samples and organoids. **E** Representative images of H&E, IHC staining (FSP1, Ki-67 and Caspase 3) and IF staining (Ki-67 and Annexin V) of OC samples and organoids. **F** The IC_50_ values (*N* = 3) of olaparib singly or combined with iFSP1 (10 μM) for 48 h in the HO-8910, SKOV3, OVCAR3 and A2780 OC cell lines. **G** The IC_50_ values (*N* = 3) of olaparib (20 μM) singly or combined with iFSP1 (25 μM) for 48 h in the 10 OC PDOs. ***P  < 0.001.

To better understand the clinical importance of FSP1 in OC, we examined FSP1 protein levels in samples from 118 OC patients in our tissue microarray by immunohistochemistry (Fig. 1A, Supplementary Fig. S1A and Table S1) and FSP1 mRNA levels in samples from our cohort of 120 OC patients by quantitative real-time PCR (qPCR) (Supplementary Fig. S1B) and found that increased FSP1 levels were associated with worse overall survival in OC patients (Fig. 1B and Supplementary Fig. S1C). Moreover, we searched a public database (https://kmplot.com/analysis/) and detected a negative correlation between the mRNA expression level of FSP1 and overall survival (Fig. 1C and Supplementary Fig. S1D). Then, as previously reported, we generated patient-derived organoids (PDOs) from specimens obtained from OC patients, representing the spectrum of OC, including high-grade serous carcinoma and clear cell carcinoma [19]. PDOs exhibited different morphological patterns, such as solid and cystic patterns, and preserved histological and phenotypic features of primary tumours, which were confirmed by the expression of various tumour markers, as shown in Fig. 1D. All established PDOs in this study were derived from BRCA-proficient patients, whose baseline data are provided in Supplementary Table S1. Based on FSP1 expression, we grouped the derived PDOs into FSP1-low (O2, O4, O6, O8 and O10) and FSP1-high (O1, O3, O5, O7 and O9) groups (Supplementary Fig. S1E and Fig. S2A). Significant overexpression of FSP1 was shown to be associated with increased levels of tumour proliferation markers (Ki-67) and low levels of apoptosis markers (Annexin V and Caspase 3) in parental tumour tissues and PDOs (Fig. 1E and Supplementary Fig. S2B). These results suggested that FSP1 was a prognostic indicator for OC patients. Next, to explore whether FSP1 inhibition could enhance the sensitivity of OC to PARPis, we compared the IC_50_ values between 4 OC cell lines treated with olaparib alone or in combination with iFSP1 (Fig. 1F) and 10 PDO models (Fig. 1G). Regardless of the sensitivity to olaparib, the IC_50_ values were significantly lower in the cotreatment group than in the olaparib monotherapy group, which demonstrated that FSP1 could be a potential strategy for sensitizing BRCA-proficient OC patients to olaparib. Hence, FSP1 overexpression was significantly associated with poor survival in human OC patients, and FSP1 inhibition might sensitize OC patients to PARPi treatment.

### FSP1 inhibition synergizes with olaparib to inhibit proliferation and increase apoptosis in BRCA-proficient OC

**Fig. 2:**
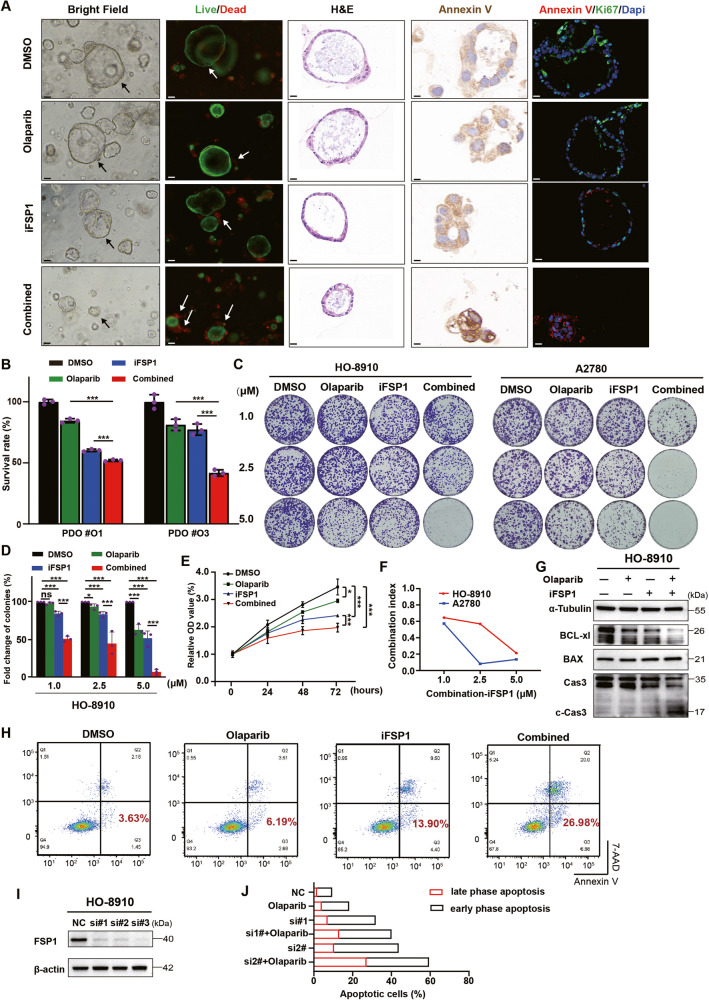
The synergistic lethality of FSP1 inhibition and Olaparib in BRCA-proficient OC. Efficacy of PDOs in response to DMSO, olaparib (20 μM), iFSP1 (25 μM) and combination for 48 h. Images of brightfield, H&E, IHC (Annexin V), live/dead staining and immunofluorescence (Annexin V and Ki-67) in the PDO. In the live/dead assay, the green represented live cells and the red was dead cells (white arrows) (**A**). PDO viability was assessed by cellTiter (**B**). ***P  < 0.001. **C**, **D** In the colony formation assay, HO-8910 and A2780 cells were treated with increasing concentrations of olaparib and iFSP1 singly and in combination for 14 days (**C**). The number and area of colonies was quantified (**D**). **E** Cell viability was measured by CCK8 assay in HO-8910 cells after olaparib (10 μM), iFSP1 (10 μM) and in combination treatment. ***P  < 0.001. **F** The combination index (CI) was calculated using the doses and response points (**D**). Immunoblots analysis of BAX, BCL-xl, pro-Caspase 3 (Cas 3) and cleaved Caspase 3 (c-Cas 3) in HO-8910 cells treated with olaparib (10 μM), iFSP1 (10 μM) and in combination for 24 h. **G** Images of flow cytometry analysis of Annexin V and 7-AAD markers in the HO-8910 cells treated with olaparib (10 μM) and iFSP1 (10 μM) singly and in combination for 24 h. **H**, **I**, **J** Response to olaparib (10 μM) treatment in FSP1-knockdown HO-8910 cells. Images of immunoblots analysis of FSP1 in the HO-8910 cells transfected with FSP1 siRNAs (**I**). Flow cytometry assessed the apoptotic cells in the HO-8910 cells in response to Olaparib (10 μM) after FSP1 knockdown (**H**, **J**).

To investigate whether FSP inhibition could enhance PARPi efficacy in OC, based on the IC_50_ values (Fig. 1G), we selected PDOs (O1 and O3) and cell lines (HO-8910 and A2780), which were insensitive to olaparib, as candidate models for further study. First, we evaluated the effects of combination treatment with olaparib and iFSP1 in PARPi-insensitive OC PDOs. By observing the morphology of the PDOs via brightfield microscopy and H&E staining, we found that the administration of any single inhibitor alone for 2 days had no effect or had little effect on the proliferation of PDOs but that the combination treatment induced a significant decrease in the expansion of PDOs. Live/dead staining also revealed that the killing effect of the combination therapy was much greater than that of any single inhibitor, which was consistent with the IF staining results for the proliferation marker Ki-67 and the apoptotic marker Annexin V (Fig. 2A). In addition, the assessment of PDO viability showed greater lethality in the combined therapy group (Fig. 2B). The synergistic effect was also tested in the HO-8910 and A2780 cell lines, which indicated that the combination of olaparib and iFSP1 caused a significant decrease in cell proliferation at low concentrations compared to either regimen alone (Fig. 2C‒E and Supplementary Fig. S3A). The combination index (CI) suggested a propensity to 0, indicating the powerful synergistic lethality of cotreatment in cancer cells (Fig. 2F). Furthermore, the percentage of apoptotic HO-8910 and A2780 cells in the combination drug group was much greater than that in the single-drug group (Fig. 2G, H and Supplementary Fig. S3B). In addition, FSP1 knockdown by siRNAs also ameliorated the efficacy of PARPis in HO-8910 and A2780 cells (Fig. 2I, J and Supplementary Fig. S3C). No obvious cell cycle arrest change was observed in the combination drug group, suggesting that cotreatment with iFSP1 and olaparib might not inhibit proliferation by inducing cell cycle arrest (Supplementary Fig. S3D, E). Taken together, these data support our hypothesis that the combination of FSP1 inhibition and olaparib treatment has synergistic effects on various preclinical models of PARPi-resistant OC.

### FSP1 inhibition sensitizes cells to PARPi treatment but not via the ferroptosis pathway

**Fig. 3:**
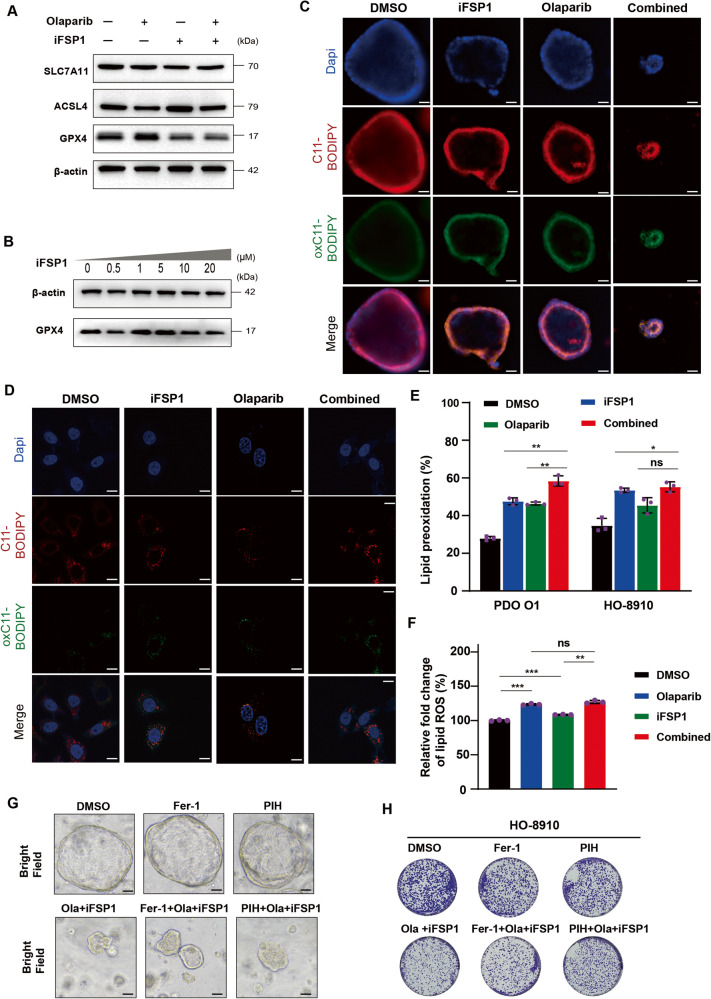
FSP1 inhibition synergizes with PARPi not via ferroptosis pathway. **A** Immunoblot analysis of SLC7A11, ACSL4 and GPX4 in the HO-8910 cells treated with DMSO, Olaparib (10 μM), iFSP1 (10 μM) and in combination for 24 h. **B** Immunoblot analysis of GPX4 in response to different concentrations of iFSP1 treatment for 24 h in HO-8910 cells. **C**‒**E** Confocal fluorescence microscopy detected lipid peroxidation in the PDO O1 (**C**) and HO-8910 cells (**D**) after treatment with DMSO, Olaparib (10 μM and 20 μM, respectively), iFSP1 (10 μM and 25 μM, respectively) and combination for 48 h. Scale bar: 20 μm and 10 μm, respectively. The percentage of lipid peroxidation was calculated in each group using the means of fluorescence (**E**). **F** The level of lipid peroxidation was quantified by flow cytometry in each group in the HO-8910 cells. **G**, **H** Two ferroptosis inhibitors, ferrostatin-1 (Fer-1) and pyridoxal isonicotinoyl hydrazone (PIH), were administrated in the cotreatment of Olaparib (20 μM and 2.5 μM, respectively) and iFSP1 (25 μM and 2.5 μM, respectively) in the PDO (**G**) and HO-8910 cells (**H**). The PDO were treated with fer-1 (5 μM) or PIH (10 μM) for 48 h (**G**). In the colony formation assay, the HO-8910 cells were treated with fer-1 (1 μM) or PIH (5 μM) for 14 days. **P  < 0.01; ***P  < 0.001; ns no significance.

Previous studies on FSP1 have focused mainly on its role in ferroptosis suppression and the regulation of tumour progression. Recently, olaparib was found to induce ferroptosis through p53 in OC [10]. Therefore, we hypothesized that combination therapy induces cell death through increasing ferroptosis in OC cells. However, immunoblot analysis demonstrated that the levels of GPX4, SLC7A11 and ASCL4 did not change more profoundly in the combination treatment group than in the single-drug treatment group; these genes are key factors involved in ferroptosis (Fig. 3A). Dose‒response analysis of GPX4 also confirmed that iFSP1 did not increase ferroptosis in OC cells (Fig. 3B). Lipid peroxidation is a major hallmark of ferroptosis; therefore, we further determined the extent of intracellular lipid ROS accumulation in the PDOs and cell lines. Indeed, lipid ROS accumulation was observed after stimulation with olaparib or iFSP1 alone in PDOs. However, only slight increases in lipid peroxidation were found in the combination group (Fig. 3C). Similarly, compared to that in the single-regimen group, cotreatment failed to induce strongly increased lipid ROS accumulation in HO-8910 cells (Fig. 3D‒F). Overall, we assumed that FSP1 inhibition did not induce cell death through promoting ferroptosis in our OC PDOs or cell lines. To verify this hypothesis, we administered two ferroptosis inhibitors, ferrostatin-1 (Fer-1) and pyridoxal isonicotinoyl hydrazone (PIH), to rescue ferroptosis-induced cell death [24, 25]. Ferrostatin-1 is a radical-trapping antioxidant [25], and PIH is an iron chelator [24] that can inhibit iFSP1-induced ferroptosis. Not surprisingly, neither of the two ferroptosis inhibitors rescued cell death in the OC-PDO model or cell lines (Fig. 3G, H), further confirming that FSP1 inhibition did not synergize with olaparib through the induction of ferroptosis in OC cells.

### FSP1 inhibition enhances olaparib efficacy by impairing NHEJ activity in BRCA-proficient OC

**Fig. 4:**
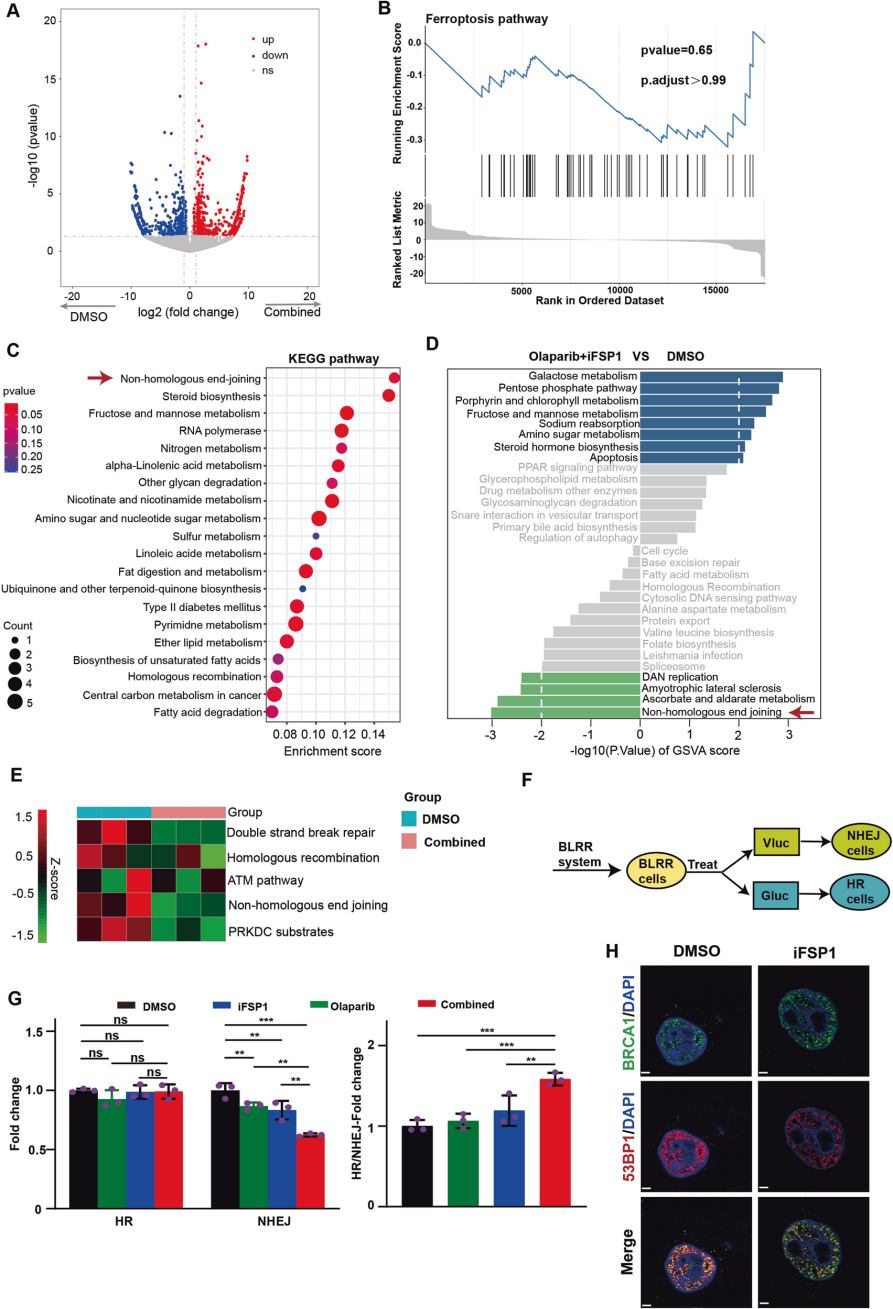
The combination of agents impairs NHEJ activity in BRCA-proficient OC. **A** Volcano plot of the results of the RNA sequence showing the differentially expressed genes (DEGs) in the PDO O1 between the control and combination treatment of Olaparib (10 μM) and iFSP1 (10 μM) (*N* = 3 per group). Upregulated and downregulated genes were in red and blue, respectively. Values were presented as the log10 of counts. **B** The GSEA enrichment of WP-Ferroptosis pathway was performed using the DEGs. **C** KEGG pathway enrichment analysis of DEGs in transcriptomes was performed. The top 20 significantly pathways were shown in the order of enrichment score. **D** The GSVA score of KEGG pathways was calculated in each group. The 15 most upregulated and downregulated pathways were shown. Values were presented as the −log10 of *p* value. **E** Heatmap of the z-score of double-strand break repair, HR, ATM pathway, NHEJ and PRKDC substrates pathways using the transcriptomes results in the RNA-seq in the control and combination treatment of Olaparib and iFSP1. **F**, **G** The BLRR cells were constructed to assess the activity of HR and NHEJ pathway in response to the treatment of DMSO, Olaparib (10 μM), iFSP1 (10 μM) and combination for 24 h in the HO-8910 cells. The fold change values of activity of HR, NHEJ and HR/NHEJ ratio in each group were shown (**G**). **P  < 0.01; ***P  < 0.001; ns no significance. **H** The nuclear levels of BRCA1 and 53BP1 after FSP1 inhibition were demonstrated by confocal fluorescence microscopy. Scale bar: 2 μm.

To further explore the mechanism by which FSP1 inhibition in combination with olaparib might increase cell death in BRCA-proficient OC, we performed RNA sequencing (RNA-seq) of PDO O1 cells treated with DMSO or combined therapy. The differentially expressed genes (DEGs) were analyzed between the two groups. A volcano plot was constructed to show the changes in the transcriptome expression of 312 downregulated and 188 upregulated genes (|log2fold change| ≥ 1, *p* value < 0.05; Fig. 4A). Unsurprisingly, gene set enrichment analysis (GSEA) indicated no significant enrichment in the ferroptosis pathway (*p* value = 0.65; Fig. 4B). To investigate the pathway through which FSP1 was inhibited, KEGG pathway enrichment analysis was subsequently performed. Interestingly, the NHEJ pathway was at the top of the list of enriched pathways, followed by steroid biosynthesis and fructose and mannose metabolism (Fig. 4C). Next, the abundance of genes associated with the KEGG pathways was evaluated by gene set variation analysis (GSVA), and a total of 8 upregulated and 4 downregulated pathways were identified. Consistent with these findings, the NHEJ pathway still ranked first among the top downregulated pathways (Fig. 4D). In addition, the Z scores revealed that the DNA repair pathway, specifically the NHEJ pathway rather than the HR pathway, was inhibited in the combined group (Fig. 4E). Therefore, we hypothesized that FSP1 participates in the NHEJ pathway and that FSP1 inhibition synergizes with olaparib through inhibiting the NHEJ pathway and increasing DNA damage. To test our hypothesis, we introduced the BLRR reporter system [23] to evaluate intracellular HR and NHEJ activities simultaneously (Fig. 4F). As expected, the activity of the NHEJ pathway decreased in the iFSP1- and olaparib-treated groups and was significantly decreased in the cotreatment group, and no obvious changes were observed in the activity of the HR pathway in any of the groups (Fig. 4G). The GSEA enrichment analysis also indicated the NHEJ pathway was significantly downregulated in the combined group (Supplementary S4A). The balance of DSB repair pathways was determined by analyzing 53BP1 and BRCA1; 53BP1 promoted the NHEJ pathway over the HR pathway, and BRCA1 promoted the HR pathway over the NHEJ pathway. We examined the levels of 53BP1 and BRCA1 and found that compared with DMSO, FSP1 inhibition resulted in less 53BP1 foci formation, with no change in the number of BRCA1 foci (Fig. 4H), which was in line with results in immunoblot analysis (Supplementary S4B, C). Taken together, these results suggested that FSP1 was indeed involved in the NHEJ pathway and that FSP1 inhibition could enhance olaparib sensitivity by suppressing NHEJ efficiency in OC.

### FSP1 involved in NHEJ by interacting with Ku70 complex

**Fig. 5:**
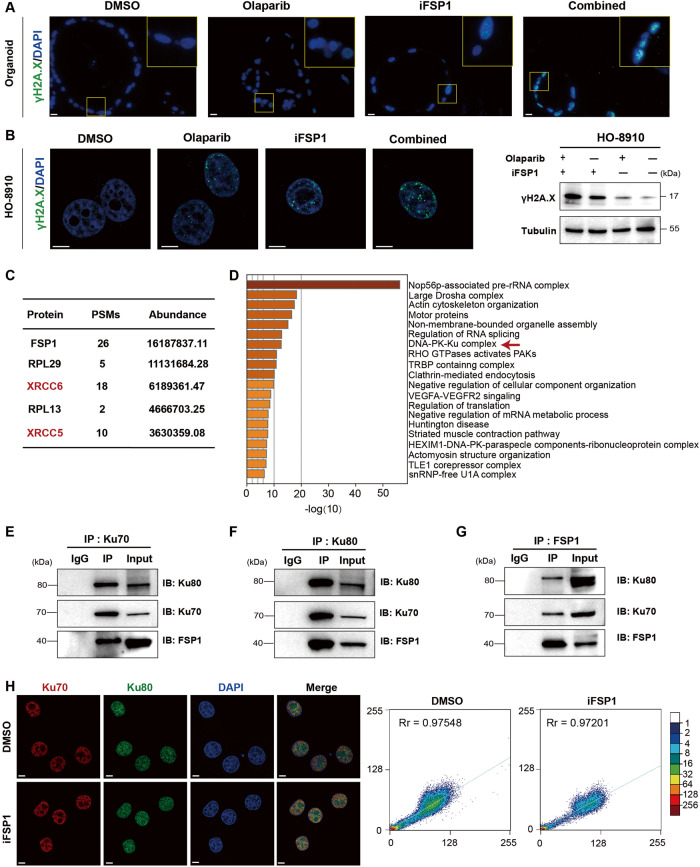
FSP1 interacts physically with Ku70/Ku80 complex. **A**, **B** Representative images of the immunofluorescent staining of γH2A.X in response to the treatment of DMSO, Olaparib, iFSP1 and in combination in the PDO (**A**) and the HO-8910 cells (**B**). Scale bar: 20 μm and 5 μm, respectively. Immunoblots analysis of γH2A.X in each group in HO-8910 cells (**B**). **C**, **D** The Co-IP with anti-FSP1 antibody in the HEK293t cells was subjected to the mass spectrometry (MS). The sorted proteins and their peptide-spectrum matches (PSMs) and abundance were listed (**C**). The enrichment analysis of the annotated proteins in the MS results was performed through Metascape (**D**). **E**‒**G** Interactions between FSP1,Ku70 and Ku80 proteins in HEK293t cells were determined by IP with IgG (control), anti-Ku70 (**E**), anti-Ku80 (**F**) or anti-FSP1 (**G**) antibody through immunoblot analysis with the indicated antibodies. **H** The nuclear colocalization of Ku70 with Ku80 protein was determined by confocal fluorescence microscopy. Scale bar: 5 μm.

NHEJ occurs throughout the cell cycle and is the major DSB repair pathway in the G1 phase, the efficiency of which is crucial for maintaining genome integrity [26]. Phosphorylated H2A.X (γH2A.X) is a hallmark of DSBs; therefore, we analyzed the expression of γH2A.X in the single-regimen or combined-regimen groups. In OC PDOs, compared to those in the DMSO group, more γH2A.X foci were observed after FSP1 inhibition or olaparib treatment alone, and the number of foci was significantly higher in the combination group, indicating that a greater number of DSBs were induced (Fig. 5A). The results in HO-8910 cells were consistent with those in PDOs (Fig. 5B). Since the role of FSP1 in the regulation of NHEJ has not been reported, it attracted our great attention. To clarify the underlying mechanism, we immunoprecipitated FSP1 and followed by mass spectrometry (MS) (Supplementary Fig. S5A, B). Interestingly, XRCC6 and XRCC5, which encode the Ku70 and Ku80 proteins, respectively were among the identified proteins with the highest abundance (Fig. 5C). Ku70 and Ku80 form a heterodimer upon DSB induction and then bind to DSB sites and serve as the docking site of the DNA-dependent protein kinase catalytic subunit (DNA-PKcs), acting as an initiator of NHEJ [27]. Then, we conducted an enrichment analysis using the identified proteins by Metascape (http://metascape.org) [28], and the DNA-PK-Ku complex pathway was at the top of the list of enriched pathways (Fig. 5D). Moreover, the physical interactions between Ku70/Ku80 and FSP1 were confirmed by IP analysis (Fig. 5E‒G). The formation of the Ku heterodimer initiates NHEJ; therefore, we assumed that FSP1 participated in NHEJ by facilitating Ku complex formation. However, neither the level of Ku70 or Ku80 nor their nuclear colocalization changed after FSP1 inhibition (Fig. 5H). Hence, we believe that FSP1 does not influence the assembly of the Ku complex at DSB ends.

### FSP1 regulates NHEJ activity through PARylation of Ku70

**Fig. 6:**
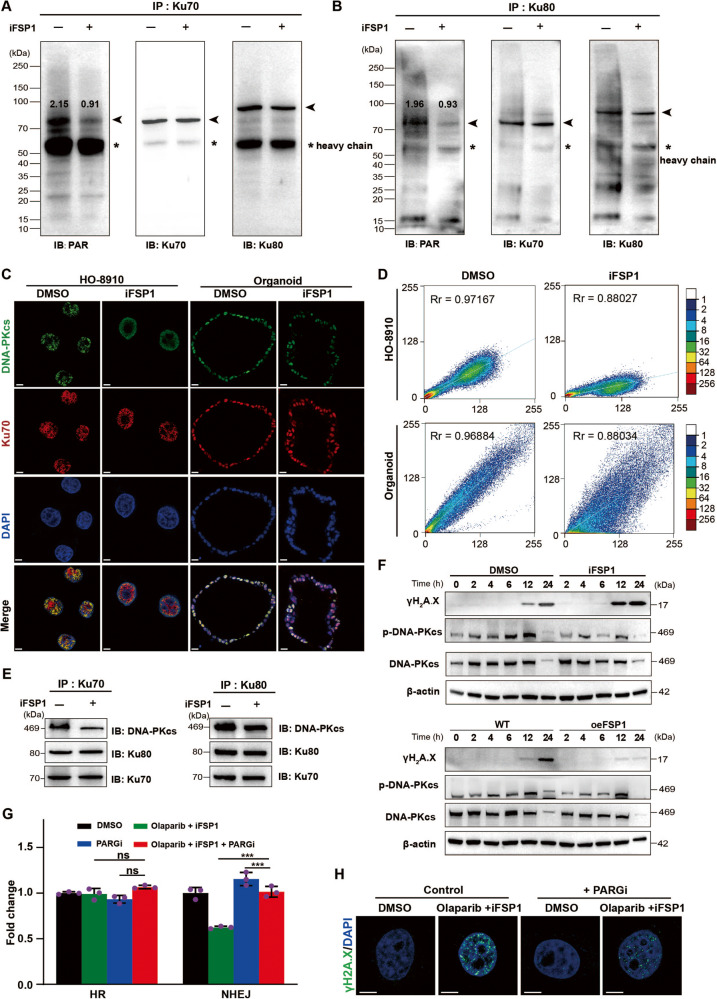
FSP1 regulates NHEJ activity through PARylation of Ku70. **A,** **B** Effect of FSP1 on PARylation of Ku70 was determined by IP with anti-Ku70 antibody (**A**) or anti-Ku80 antibody (**B**) and immunoblots analysis in HEK293t cells. The scaled quantification of PARylation were shown. *: heavy chain. **C**‒**E** Immunofluorescence (**C**) and immunoblots analysis (**E**) determined ternary complex formation of DNA-PKcs with Ku70/Ku80 complex after FSP1 inhibition in the HO-8910 cells or PDOs. The Pearson’s correlation coefficient of colocalization of Ku70 and DNA-PKcs was calculated (**D**). Scale bar: 2 μm and 20 μm, respectively. **F** The immunoblots analysis determined the levels of γH2A.X, DNA-PKcs and phosphate-DNA-PKcs (p-DNA-PKcs) in the HO-8910 cells to the treatment of FSP1 inhibition or overexpressing FSP1. **G**, **H** The PARG inhibitor PDD00017273 (PARGi) was used to avoid the PARylation degradation in the HO-8910 cells. The HR and NHEJ activity were determined by the BLRR system (**G**). ***P  < 0.001; ns no significance. The immunofluorescence showed the levels of γH2A.X in each group (**H**). Scale bar: 5 μm.

As an early response to DSBs, the Ku complex is recruited to DSB sites to initiate NHEJ, the efficiency of which relies on Ku70 PARylation [29, 30]. Olaparib is a selective inhibitor of PARP1/2 and thus inhibits downstream PARylation. As FSP1 did not influence the expression of Ku70/Ku80 or their binding capacity, we investigated whether FSP1 affects the PARylation of Ku70/Ku80. Thus, we analyzed the PARylation level of Ku70/Ku80 after FSP1 inhibition. Immunoblot analysis revealed that the Ku70 protein, but not the Ku80 protein, could be modified by PARylation (Fig. 6A, B; Supplementary Fig. S6A, B) and that its expression was strongly reduced after FSP1 inhibition (Fig. 6B), revealing the novel role of FSP1 in regulating NHEJ through the PARylation of Ku70. The Ku complex recruits DNA-PKcs and forms the DNA-PKcs/Ku complex at DSBs, which is the key component of the NHEJ process [31]. Since FSP1 did not regulate the interaction between Ku70 and Ku80, we hypothesized that FSP1 affects the assembly of the DNA-PKcs/Ku complex. By IF, we found that DNA-PKcs was mostly localized in the nucleus in both the PDO and HO-8910 models. Colocalization analysis revealed that less DNA-PKcs bound to Ku70 in the FSP1 inhibition group than in the DMSO group (Fig. 6C, D; Supplementary Fig. S6C, D). Co-IP analysis further confirmed that FSP1 inhibition disrupted the interaction between DNA-PKcs and Ku70/Ku80 (Fig. 6E). The activity of DNA-PKcs can be modulated by the autophosphorylation of PQR/2056 clusters [31]. As expected, after FSP1 was inhibited by iFSP1 or silenced by siRNA, the phosphorylation of DNA-PKcs was attenuated, and the expression of γH2A.X was subsequently increased (Fig. 6F and Supplementary Fig. S6E‒G); moreover, FSP1 overexpression partly rescued γH2A.X levels (Fig. 6F and Supplementary Fig. S6H). Poly ADP-ribose glycohydrolase (PARG) is the major enzyme involved in the degradation of PARylation products generated by PARPs. We used the PARG inhibitor (PARGi) PDD00031705 to prevent PARylation removal from Ku70. As expected, PDD00031705 successfully restored NHEJ activity and reduced DSBs in the cotreatment group (Fig. 6I, H and Supplementary Fig. S6I). Overall, FSP1 inhibition impaired NHEJ through disrupting the interaction between DNA-PKcs and Ku70/Ku80, which was dependent on the PARylation of Ku70.

### FSP1 inhibition and olaparib treatment have synergistic effects in vivo

**Fig. 7:**
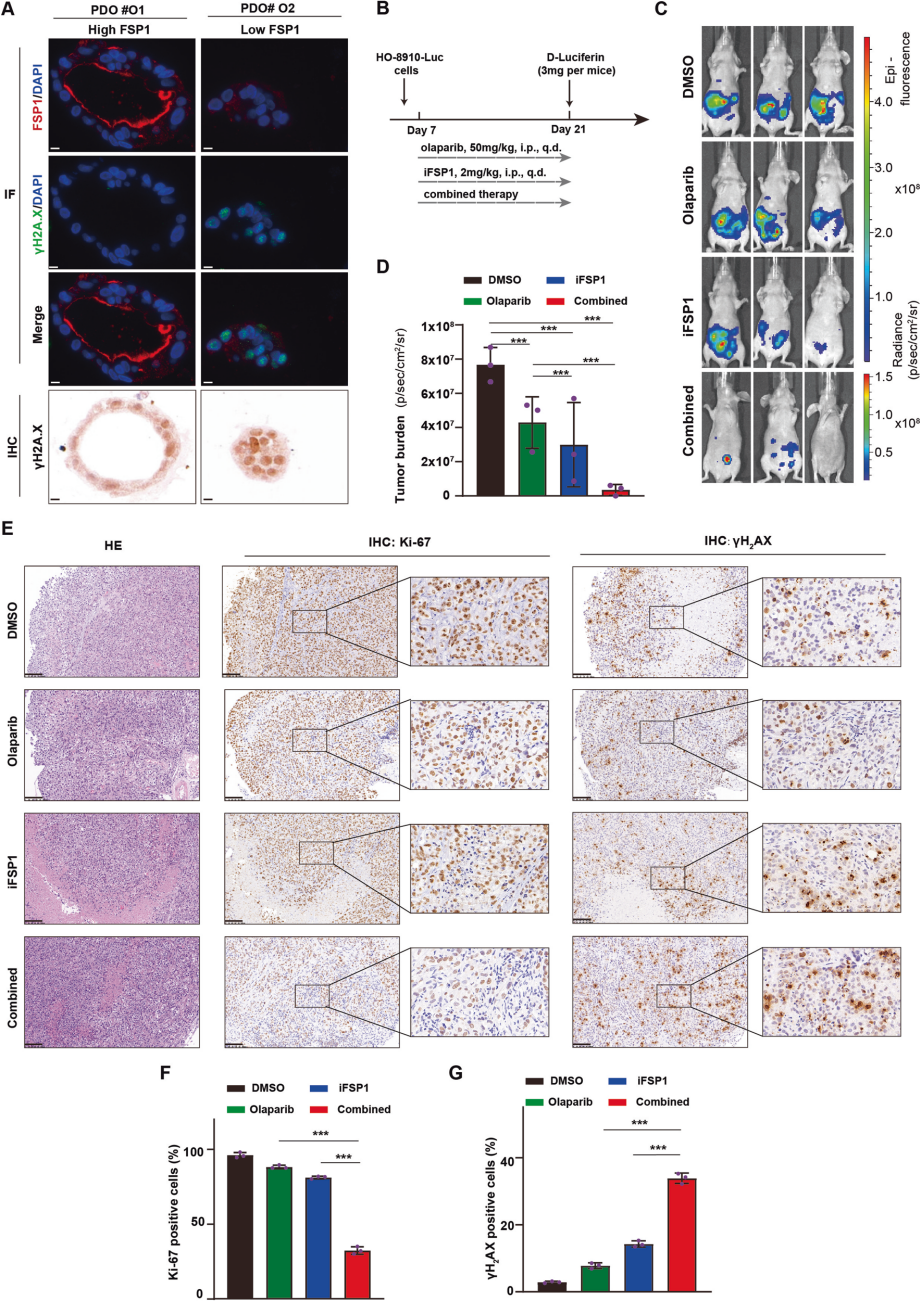
FSP1 inhibition and Olaparib show synergistic killing effects in vivo. **A** Representative images of immunofluorescence and IHC staining of γH2A.X in the FSP1-high and FSP1-low PDOs. Scale bar: 10 μm. **B**‒**D** The HO-8910 cells was injected into the abdominal cavity of BALB/c mice, and then treated with DMSO, Olaparib (50 mg/kg/d), iFSP1 (2 mg/kg/d) and in combination for 14 days (**B**). Representative bioluminescent images of mice in the four groups were taken on days 21 (*N* = 3 per group) (**C**). The tumour burden was calculated using the luminescence values (**D**). ***P  < 0.001. **E**‒**G** Representative images of H&E and IHC staining of Ki-67 and γH2A.X in the tumours harvested from each group (**E**). The Ki-67 positive cells (**F**) and γH2A.X positive cells (**G**) were calculated and shown as a histogram. Scale bar: 100 μm. Data were means ± SD. ***P  < 0.001.

To further determine the role of FSP1 in maintaining genome integrity, we explored the association between the levels of γH2A.X and FSP1 in PDOs. According to the IF analysis, FSP1 localized to the nucleus, cytoplasm and cell membrane. Notably, FSP1 was abundant in the PDOs with cystic structures and grew faster than in those with solid structures. Subsequently, more γH2A.X foci were observed in the FSP1-low PDOs than in the FSP1-high PDOs (Fig. 7A), indicating that FSP1-regulated NHEJ was crucial for maintaining genome integrity. To explore the synergistic efficacy of the combinations of these agents in vivo, we assessed the effects of a single dose or a combination of iFSP1 and olaparib (50 mg/kg/day) in a xenograft mouse model (Fig. 7B). After 14 days of treatment, the tumour burden in the iFSP1 group was lower than that in the DMSO group; of note, the tumour burden was significantly lower in the combination group than in any single group (Fig. 7C, D), a finding that was also supported by the Ki-67 staining of the tumour sections in each group (Fig. 7E, F). Moreover, more γH2A.X foci were induced in the cotreatment group than in the monotherapy group (Fig. 7E, G). Furthermore, no significant changes were observed in the heart, liver, lung, spleen, or kidney, suggesting that the therapy was safe (Supplementary Fig. S7).

## Discussion

There are three main findings of this study. First, we illustrate that FSP1 expression is associated with sensitivity to PARPi treatment in BRCA-proficient OC cells. Second, FSP1 is found for the first time to enhance NHEJ activity via nonferroptosis mechanisms, in which FSP1 promotes the recruitment of DNA-PKcs to Ku70/Ku80 at DSBs through stimulating the PARylation of the Ku70 protein. Finally, FSP1 inhibition exerts synergistic lethal effects with olaparib in multiple OC models without increasing toxic side effects, suggesting that this combined approach is a promising strategy for sensitizing OC patients to olaparib treatment and improving patient prognosis.

FSP1 (AIFM2) has been found to be a key player in the protective pathways against ferroptosis [12, 13]. FSP1 mainly localizes to the plasma membrane and acts as an antioxidant dependent on NADPH and NADH, regardless of intracellular glutathione levels. Because of its role in ferroptosis, strategies targeting FSP1 have been developed, and iFSP1 is the first potent inhibitor that has been shown to suppress cell proliferation and induce ferroptosis in vitro and in PDX models [18]. In the present study, treatment with iFSP1 alone or in combination with olaparib indeed effectively induced cell death in OC; however, these killing effects could not be reversed by ferroptosis inhibitors, which indicates other underlying functions of FSP1. FSP1 was initially identified as a member of the mitochondrial protein apoptosis-inducing factor (AIF) family; however, unlike AIF, FSP1 also localizes to the cytoplasm because of a lack of mitochondrial localization signals [32]. Apart from acting as an oxidoreductase, FSP1 can also bind DNA, leading to conformational changes in FSP1 [33]. Moreover, the DNA-binding domain competes with NADPH, which is likely the reason why the oxidoreductase activity of FSP1 is diminished after the translocation of FSP1 from the mitochondria to the nucleus [32]. However, the molecular processes involved in DNA binding to the FSP1 protein are unknown. In this study, for the first time, we revealed the participation of FSP1 in the process of the PARylation of the Ku70 protein, followed by DNA-PKcs recruitment and subsequent DSB repair. DNA-PKcs acts as a sensor of DNA damage and is recruited to DSB sites to form the DNA-PKcs/Ku70/Ku80 complex. Dysfunction in this complex has been reported to be associated with poor outcomes in patients with various types of solid tumours. Previous studies have shown that targeting DNA-PKcs can sensitize tumour cells to DNA damage drugs and prolong their proliferation [31, 34]. Our data suggest that the interaction of DNA-PKcs with the Ku70/Ku80 complex requires PARylated Ku70 instead of PARylated Ku80, a process that is mediated by the FSP1 protein. As a result, FSP1 inhibition by iFSP1 can disturb the assembly and activation of the DNA-PKcs/Ku70/Ku80 complex and thus decrease NHEJ activity, making it a therapeutic candidate for reversing PARPi resistance in OC. Hence, we investigated the undefined role of FSP1 in DSB repair beyond ferroptosis protection; however, the mechanisms determining the selection and communication between these two pathways need to be further explored.

The most studied mechanisms of acquired PARPi resistance include the restoration of HR activity due to reverse mutations in HR genes, the stabilization of replication forks induced by other DNA repair factors and the reduction in the effective intracellular dose of PARPis due to the increased rate of PARPi removal [6, 8, 35]. However, the cause of primary drug resistance has not been elucidated. In the present study, we revealed that inhibiting FSP1 and downstream Ku70 PARylation might be potential strategies for enhancing PARPi efficacy in BRCA-proficient OC. PARylation is a reversible and transient posttranslational modification that occurs upon DNA damage, and the occurrence and degradation of PARylation are catalyzed by PARP enzymes and PAR glycohydrolase (PARG), respectively. Evidence shows that the restoration of PARylation caused by PARG depletion can stabilize the replication fork and promote downstream DNA repair components, thus leading to PARPi resistance [36, 37]. This finding is in line with our data, which showed that increased levels of FSP1 promoted the PARylation of Ku70 and facilitated the recruitment of DNA-PKcs to complete DNA repair, thereby reducing sensitivity to PARPis. On the other hand, genome integrity is needed for sensitivity to PARPis [8]. The DNA-PKcs/Ku70/Ku80 complex activates the NHEJ pathway, which is the dominant mechanism of DSB repair. Fluctuations in PARylated Ku70 levels result in alterations in NHEJ activity and therefore cellular genome instability, which provides another explanation for FSP1-mediated PARPi resistance. However, further studies are needed to explore whether FSP1 is associated with the PARylation of other target proteins involved in other cellular processes, such as mitosis, which also requires PARylation. HR activity did not increase significantly when NHEJ activity was impaired by FSP1 inhibition, which we suppose might be because FSP1 is also responsible for spindle formation or chromosome segregation and eventually interrupts mitosis [38]. Collectively, our data provide new insights into PARPi resistance, suggesting new therapeutic strategies to overcome drug resistance in OC.

Currently, BRCA1/2 mutations are the most established biomarkers for PARPi resistance, and germline and tumour testing are recommended for OC patients at diagnosis. Nevertheless, increasing evidence has shown that PARPi resistance is still present in patients without BRCA1/2 mutations; thus, massive efforts have been made to investigate other potential markers for predicting the sensitivity of PARPis. Among these genes, those involved in HR pathways, such as RAD51, ATM and ATR, are the most explored candidates [39, 40]. Preclinical studies have shown that the expression of these genes is positively correlated with sensitivity to PARPi treatment and can enhance the response to PARPi treatment in cancer cells. However, several clinical trials have shown that the accuracy of these genes in predicting the response to PARPis is similar to that of BRCA1/2 in patients with recurrent ovarian cancer and even lessened when olaparib is combined with bevacizumab in newly diagnosed patients [41, 42]. This unsatisfactory prognosis requires urgent efforts to identify additional potential markers. In the present study, FSP1 overexpression was shown to be associated with a poor response to PARPis, and FSP1 inhibition further ameliorated the effects of PARPis in OC cell lines and PDOs, suggesting that FSP1 is a promising biomarker for predicting the response to PARPis in OC. Notably, pharmaceutical drugs targeting FSP1 are now being developed. iFSP1 is one of the first-generation inhibitors reported to regulate human FSP1 through binding to residue F360 within human FSP1 and showed encouraging efficacy in inducing cell death in our study [43]. icFSP1 is the latest generation of inhibitors that induces the subcellular translocation of FSP1 from the membrane and has cytotoxic effects on cancer cells [9]. Taken together, these findings suggest that FSP1 is a potential biomarker for the prediction of the PARPi response and provide a rationale for overcoming PARPi resistance in OC.

In summary, our study demonstrated a novel role of FSP1 in DSB repair in addition to its ability to protect against ferroptosis. An increase in the level of FSP1 in OC could alter NHEJ activity and consequently genome instability, contributing to resistance to PARPis. FSP1 inhibition also promoted vulnerability to PARPis in BRCA-proficient OC patients, providing new insight into sensitizing patients to PARPi treatment.

### Supplementary information

original data files

Supplementary_information

Table S1

Table S2

Table S3

## Data Availability

The raw sequence data reported in this paper have been deposited in the Genome Sequence Archive (Genomics, Proteomics & Bioinformatics 2021) in National Genomics Data Center (Nucleic Acids Res 2022), China National Center for Bioinformation/Beijing Institute of Genomics, Chinese Academy of Sciences (GSA-Human: HRA005138) that are publicly accessible at https://ngdc.cncb.ac.cn/gsa-human.
