# Supplementary material for: FSP1 inhibition enhances olaparib sensitivity in BRCA-proficient ovarian cancer patients via a nonferroptosis mechanism
Source: Cell Death Differ. 2024 Feb 19;31(4):497–510. doi: 10.1038/s41418-024-01263-z (PMC11043371; doi:10.1038/s41418-024-01263-z)

Figure 2G

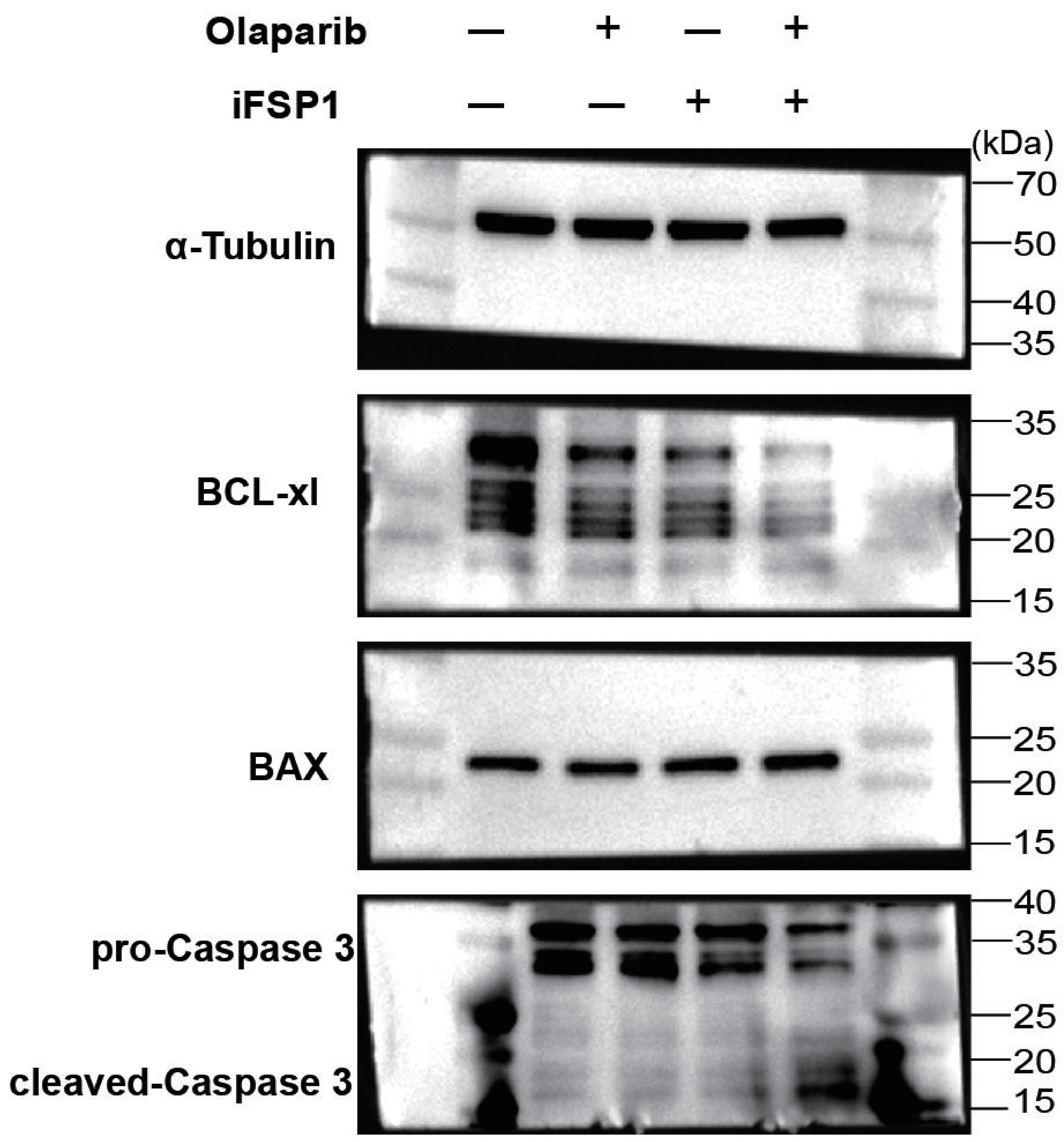

Figure 2I

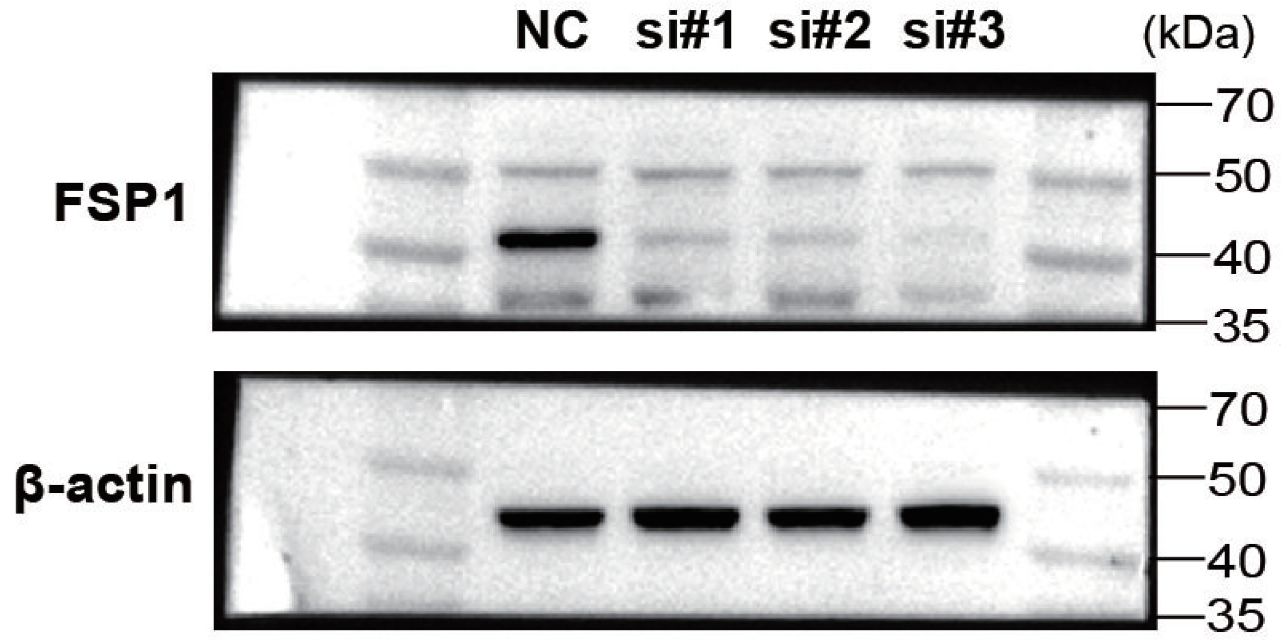

Figure 3A

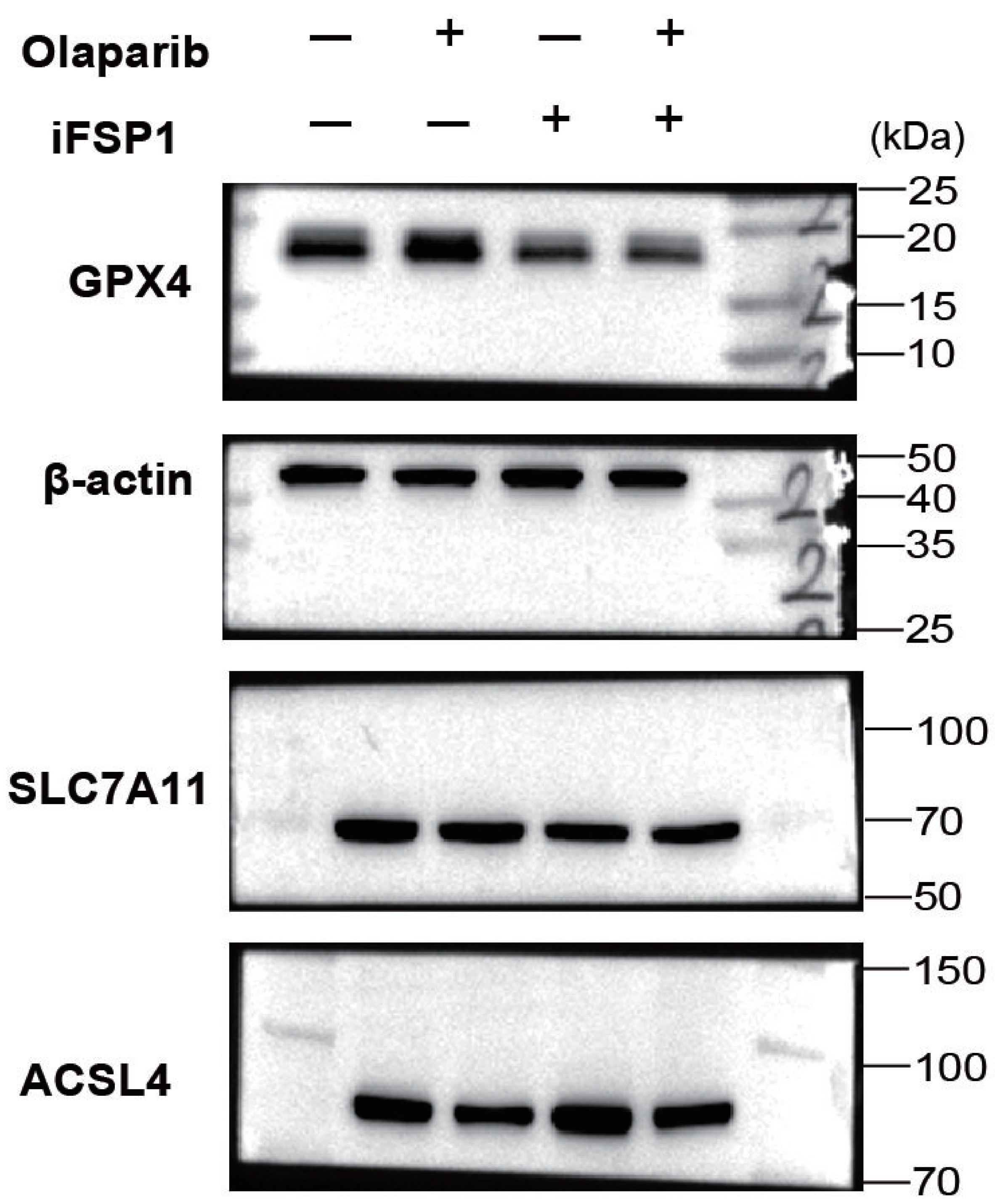

Figure 3B

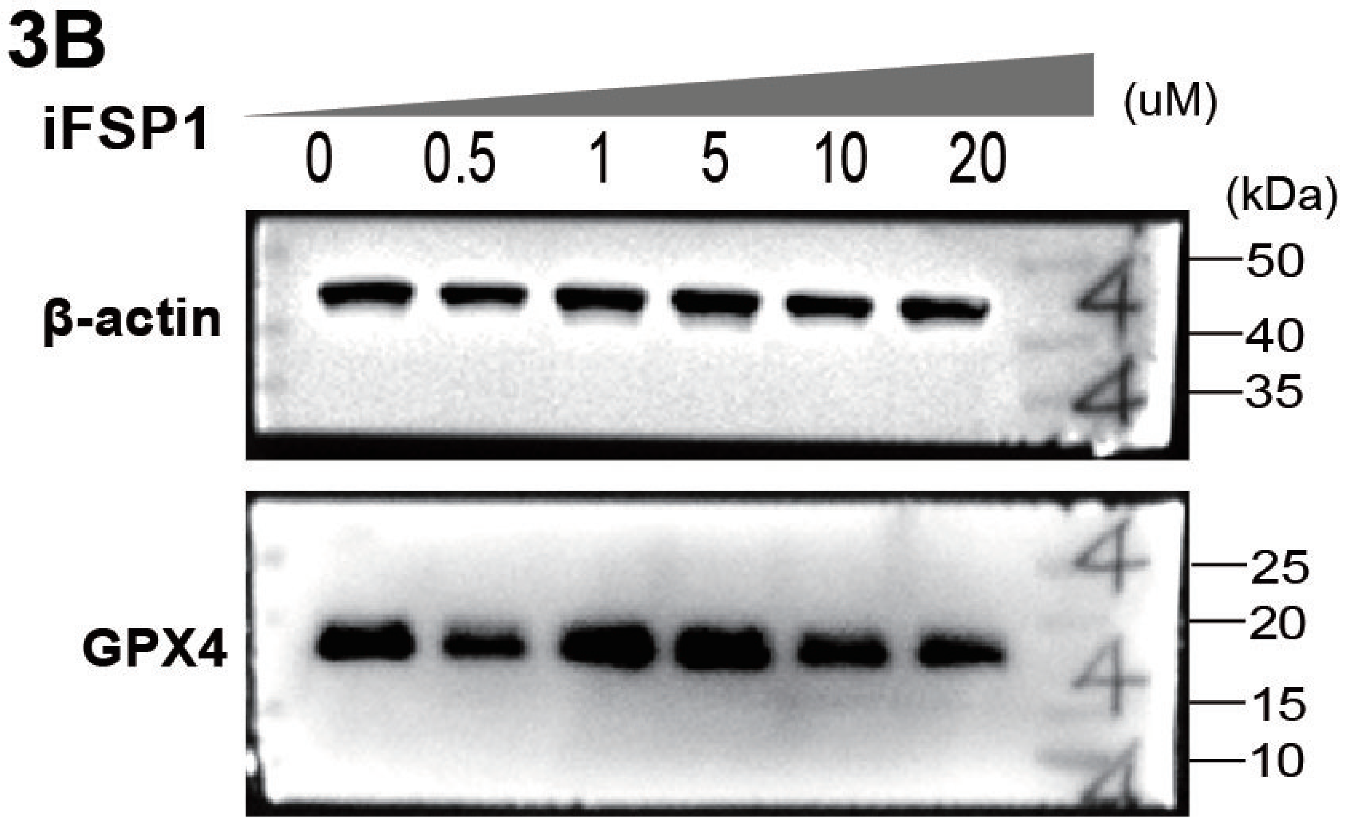

Figure 5A

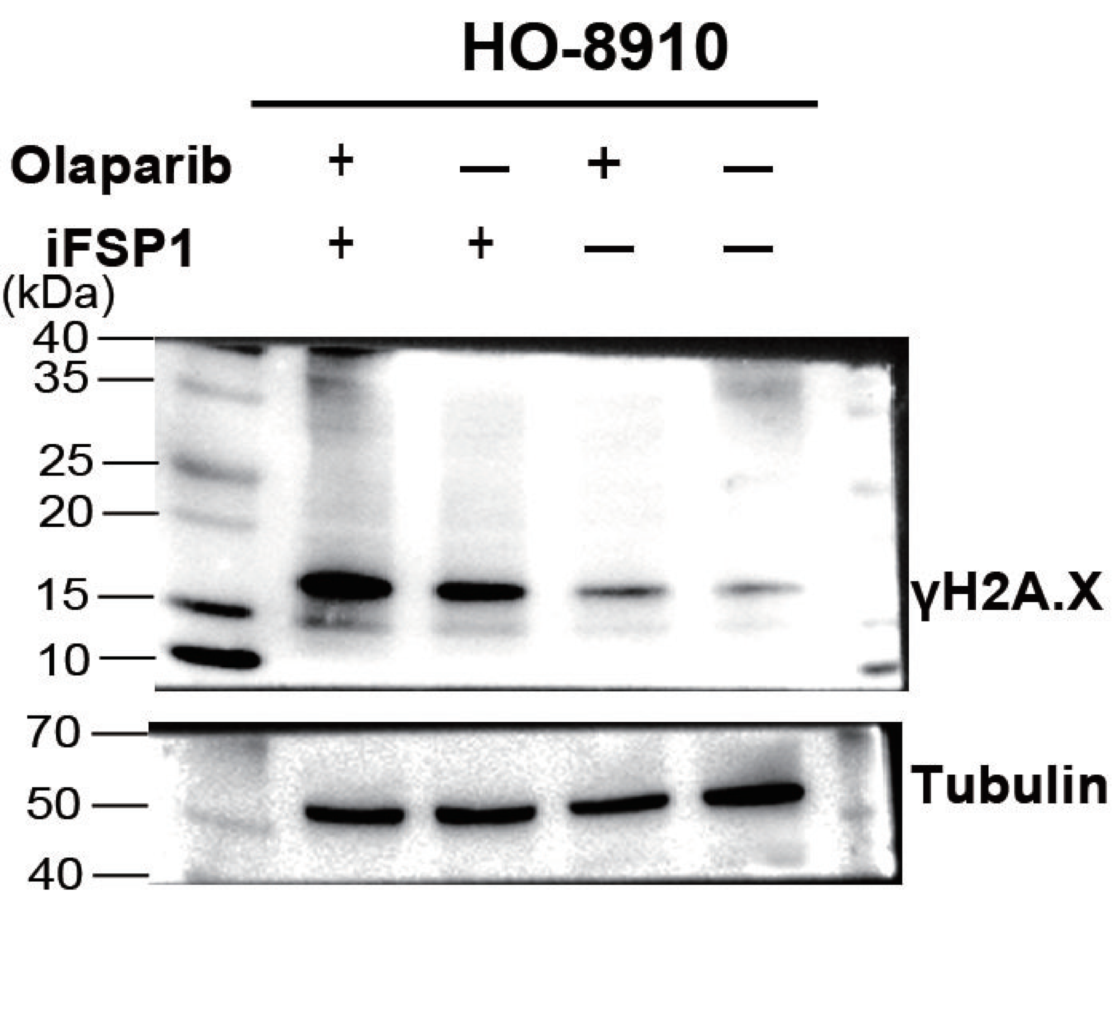

Figure 5E

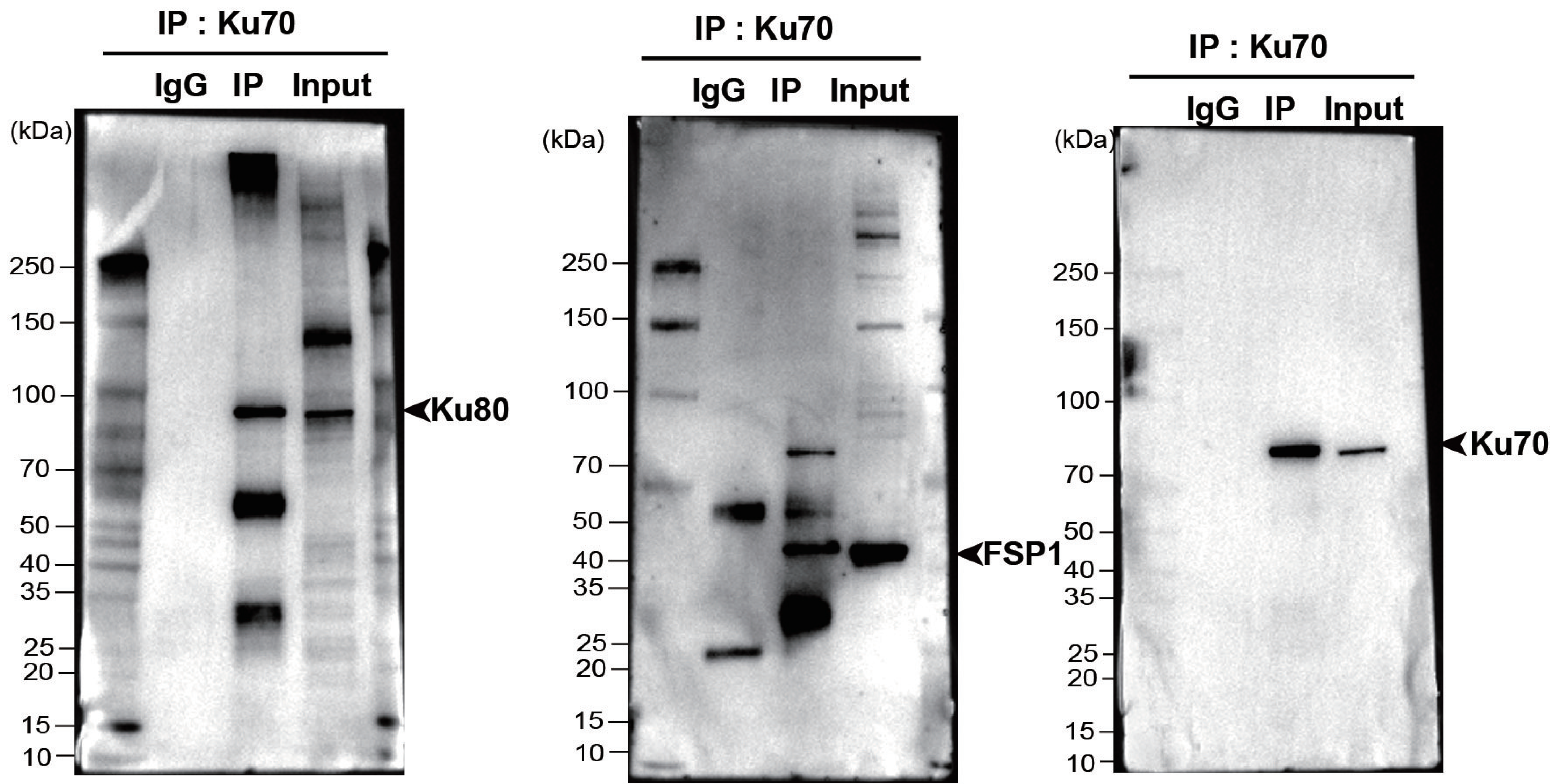

Figure 5F

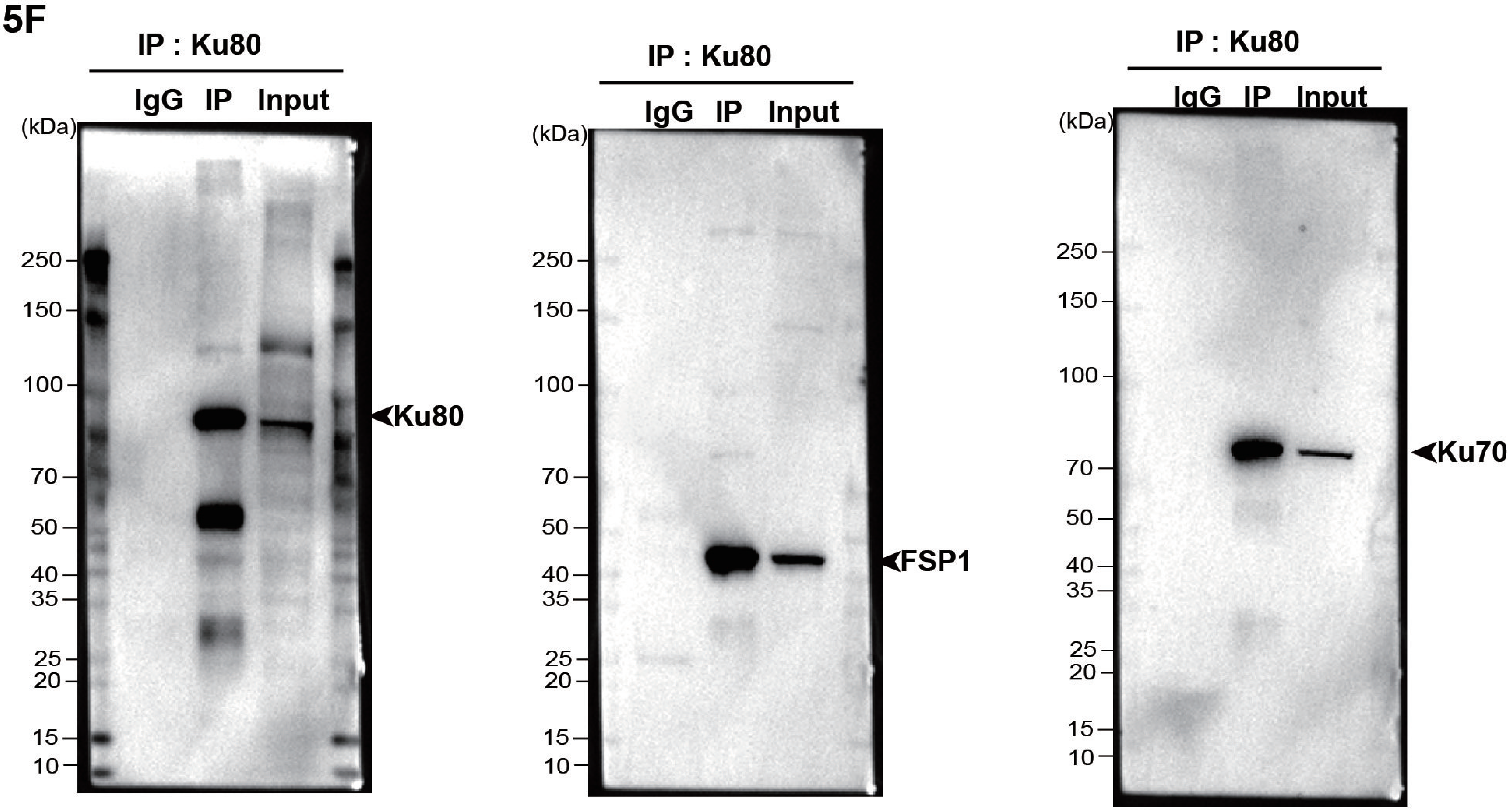

Figure 5G

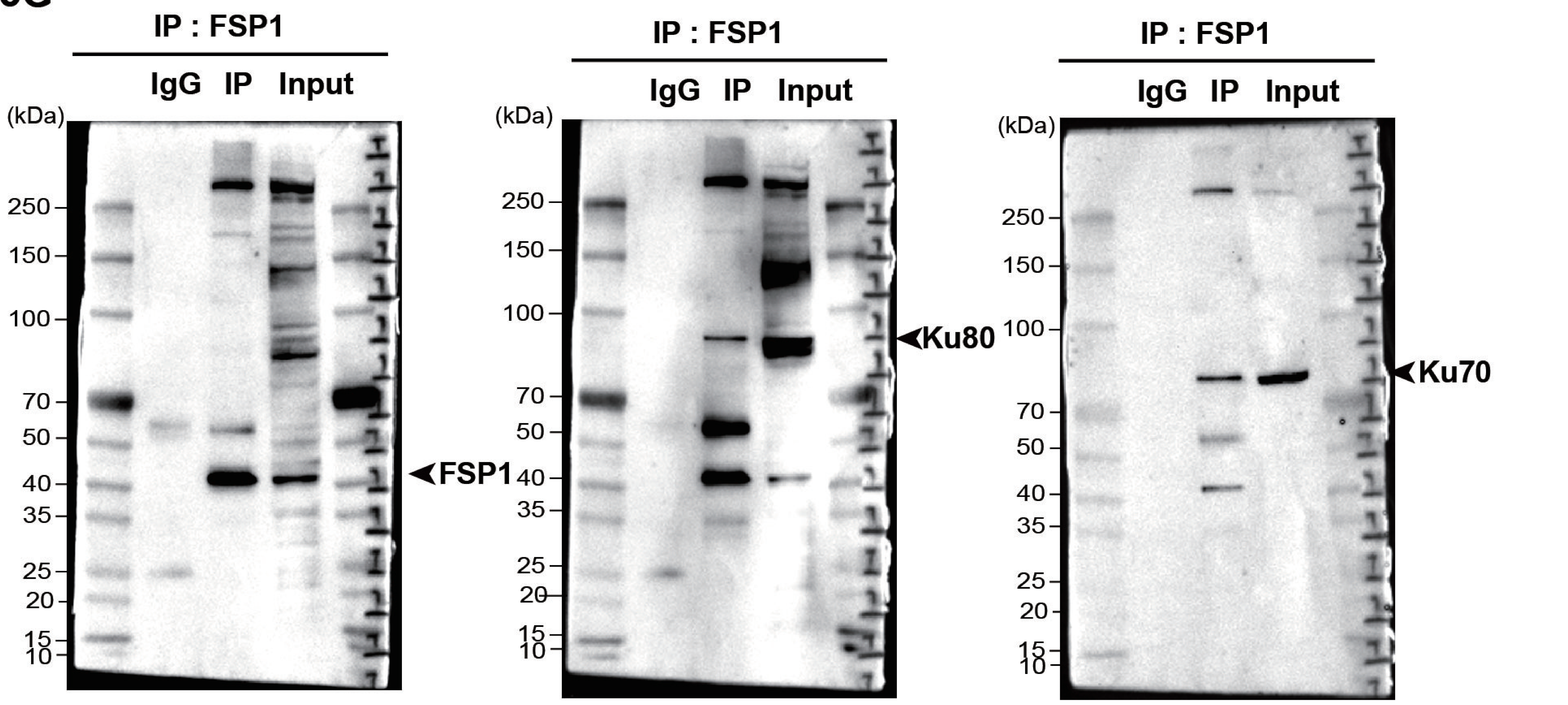

Figure 6A

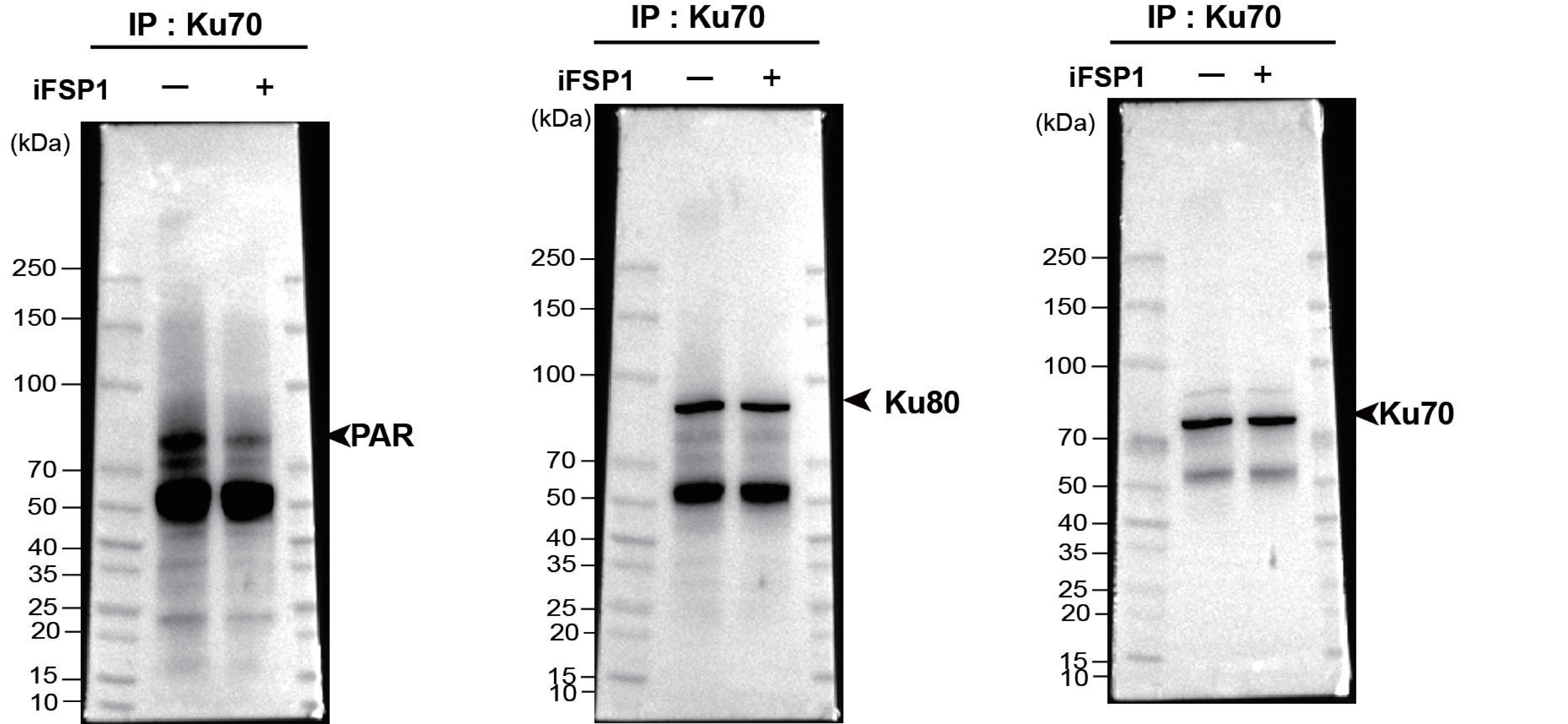

Figure 6B

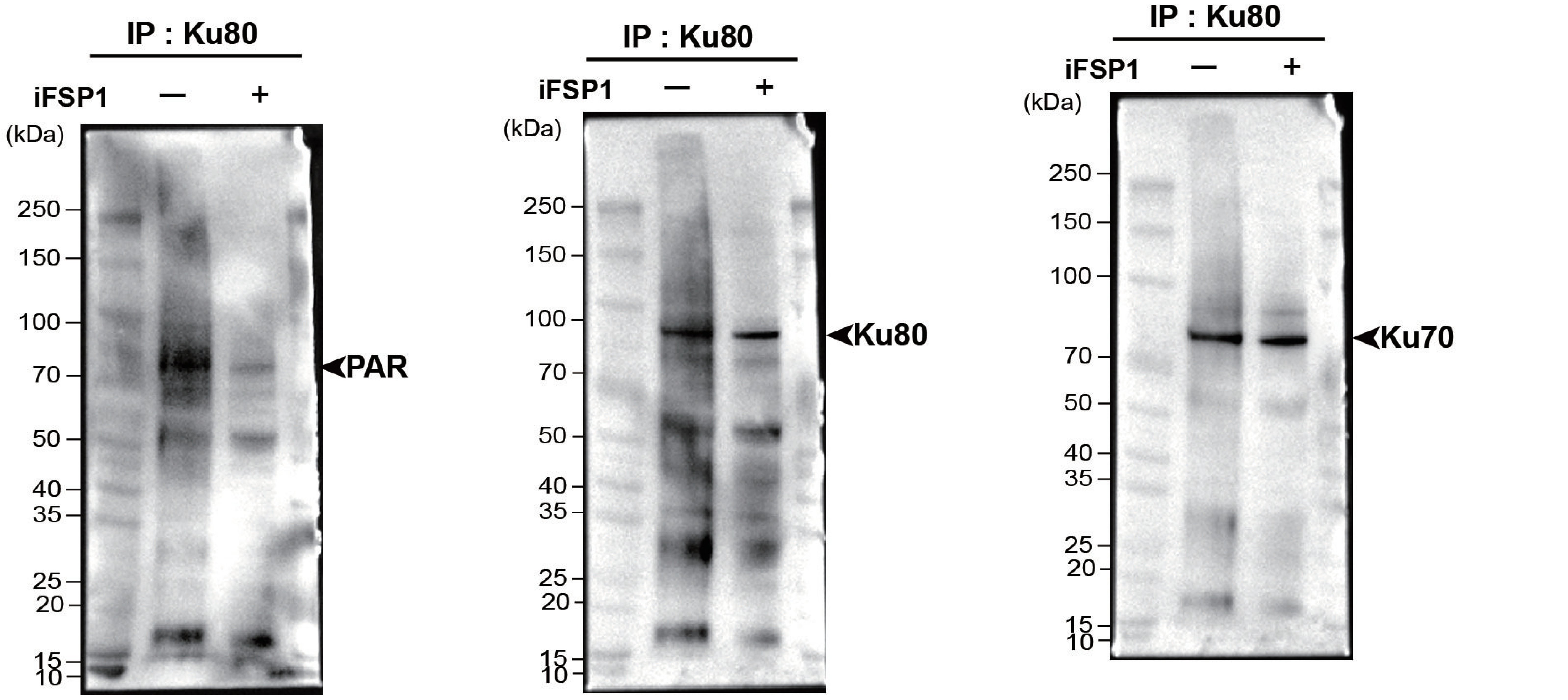

Figure 6E

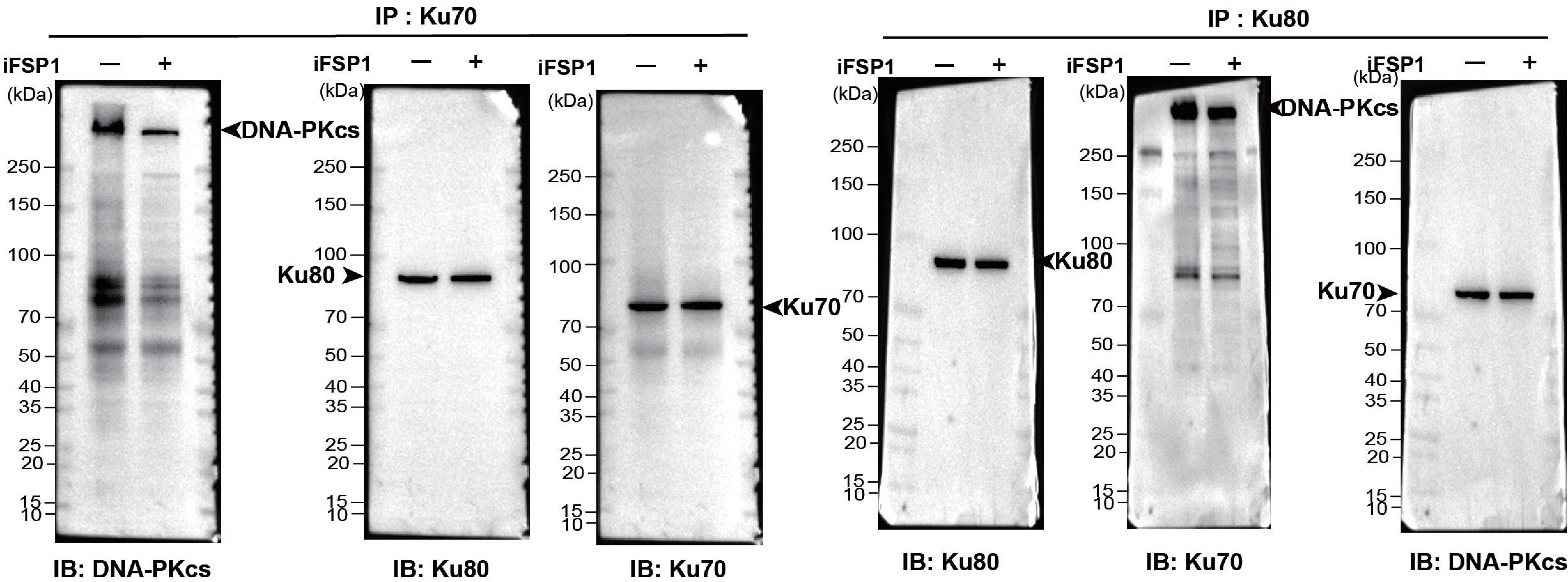

Figure 6F

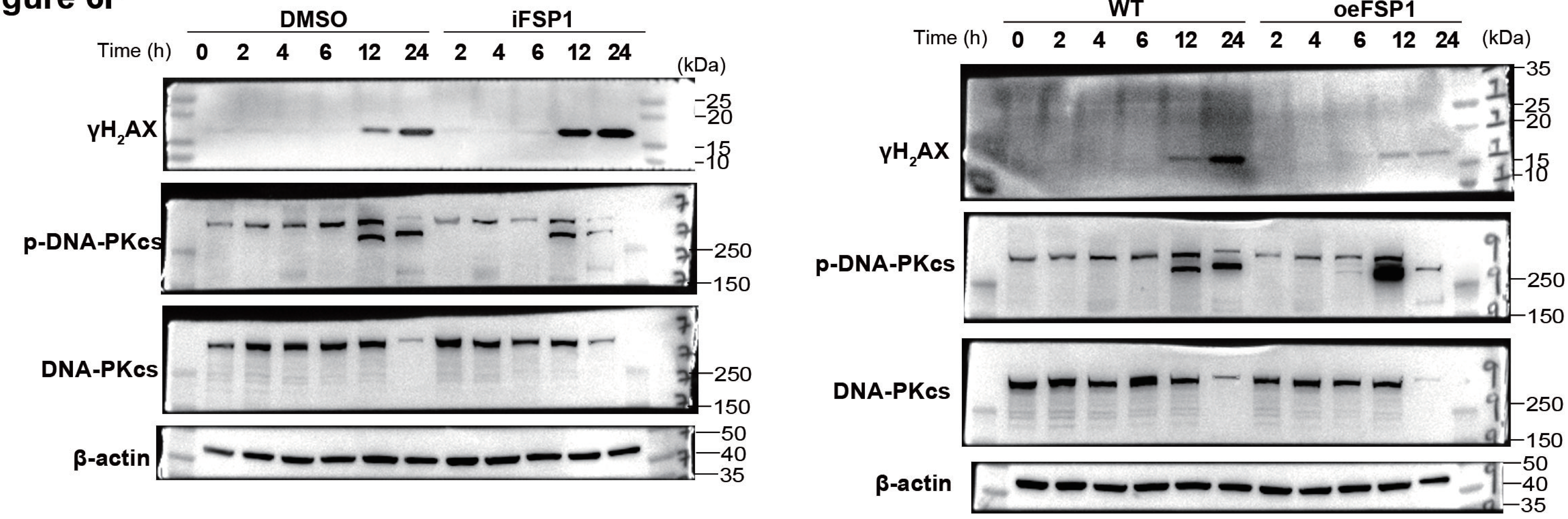

Figure S1E

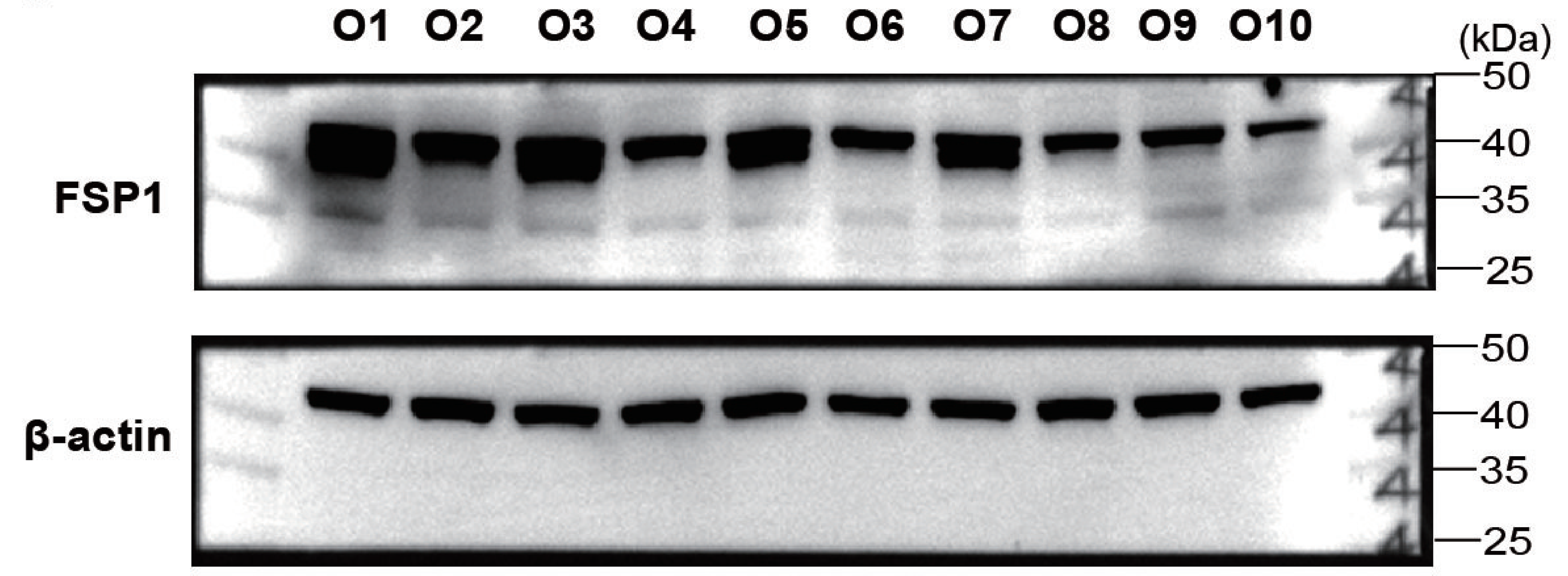

Figure S2B

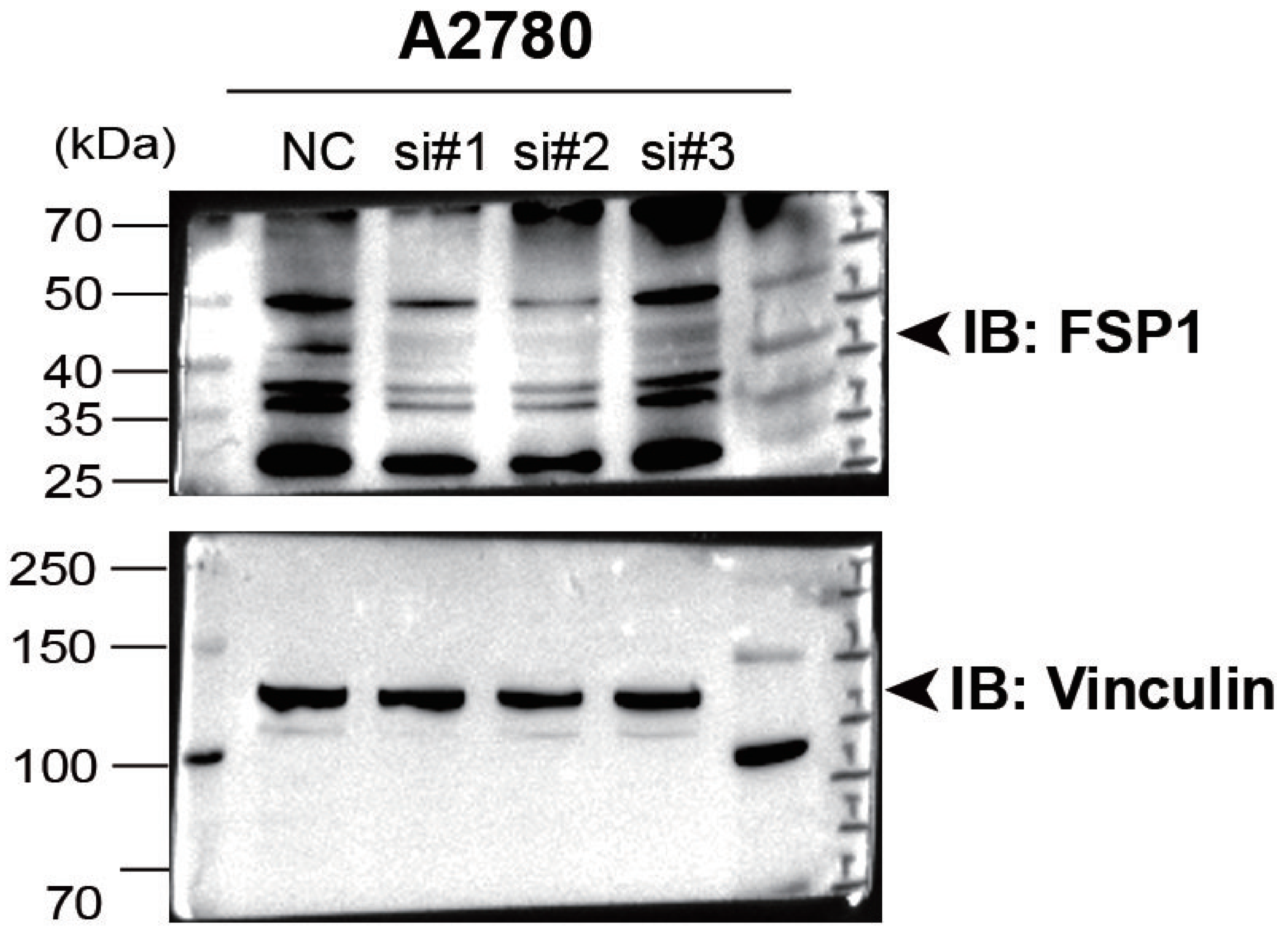

Figure S3A

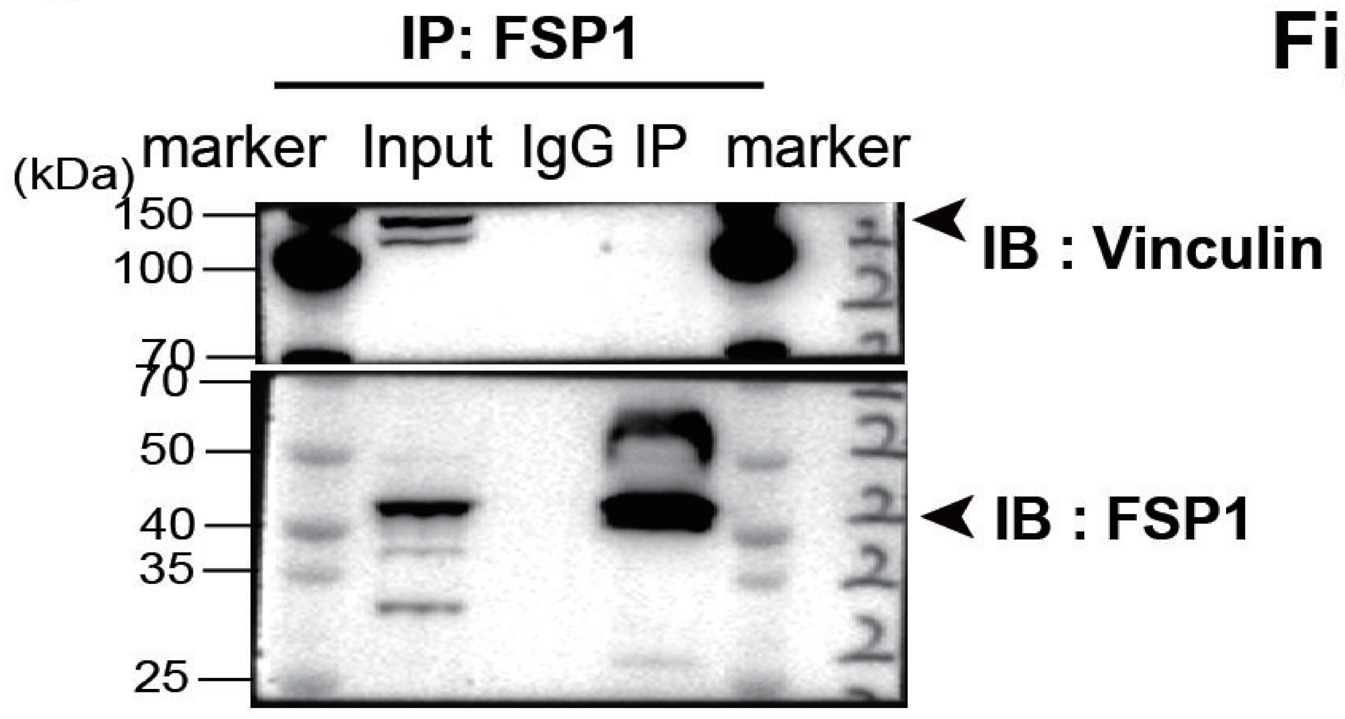

Figure S4B

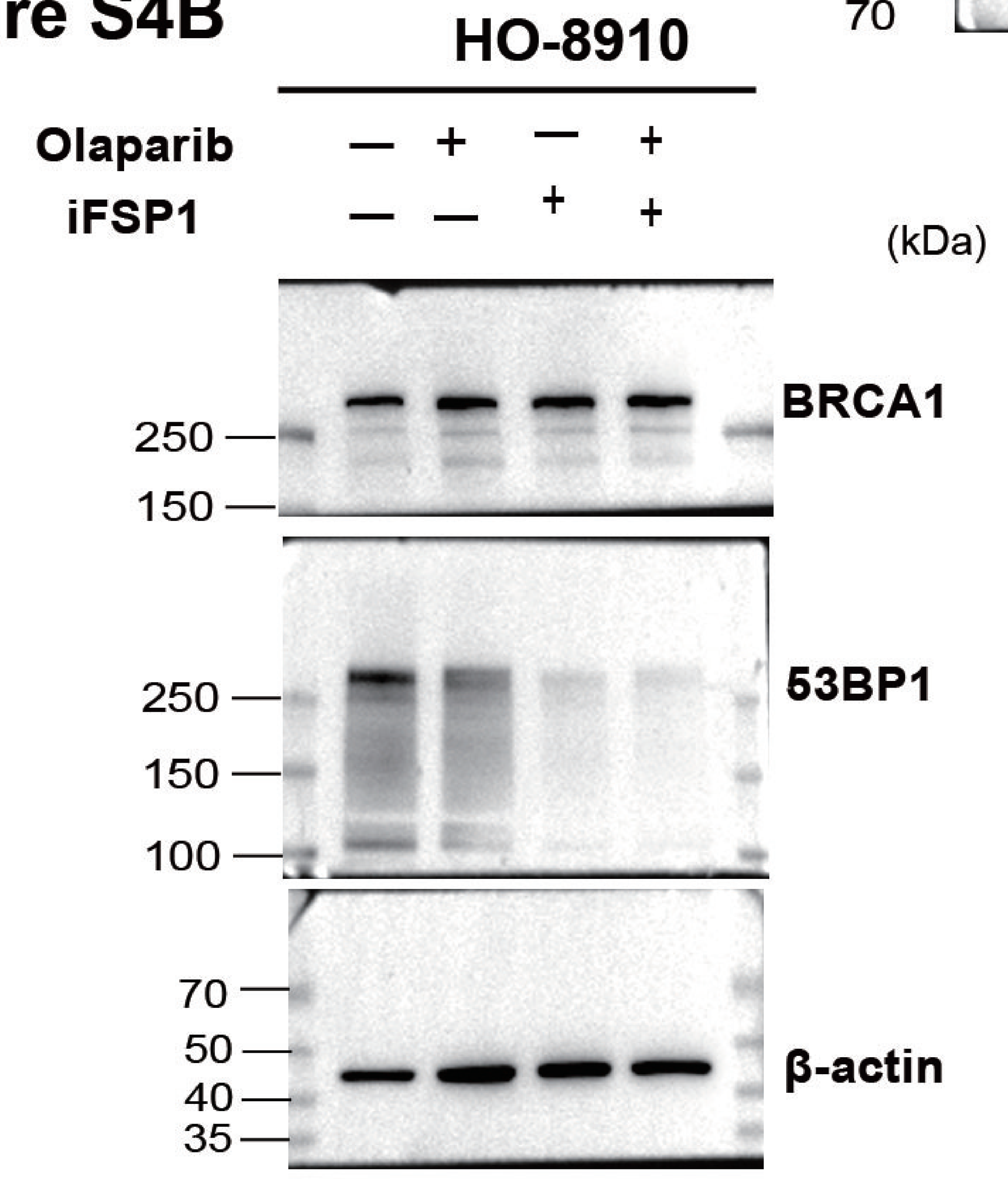

Figure S6A

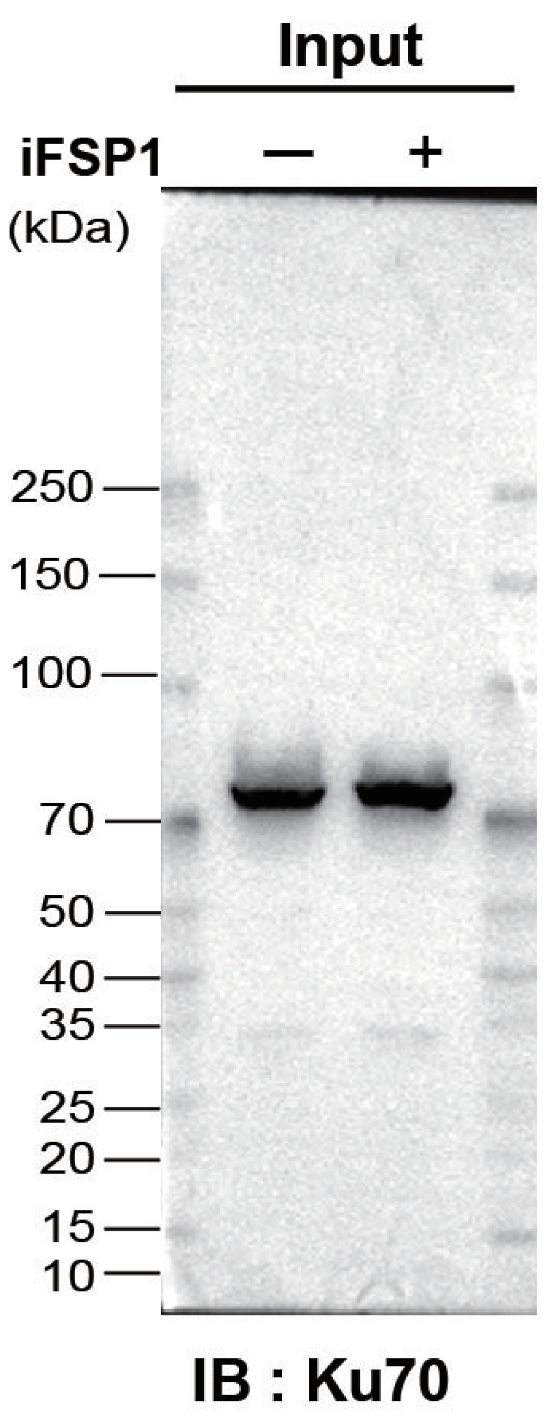

Figure S6B

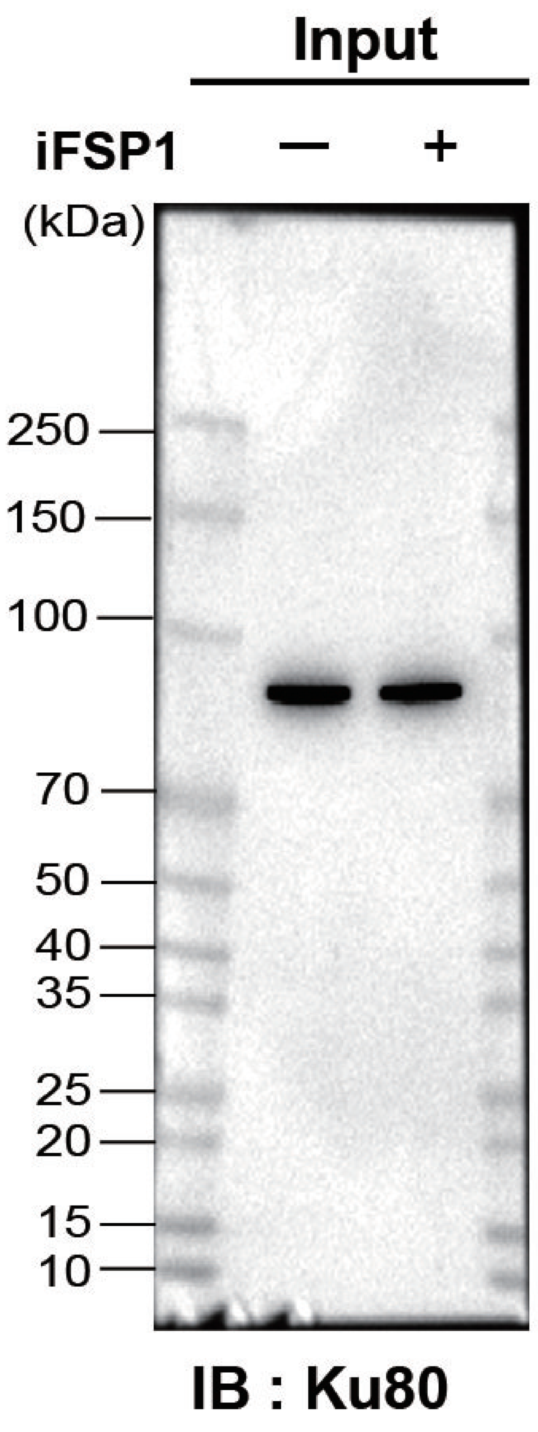

Figure S6C

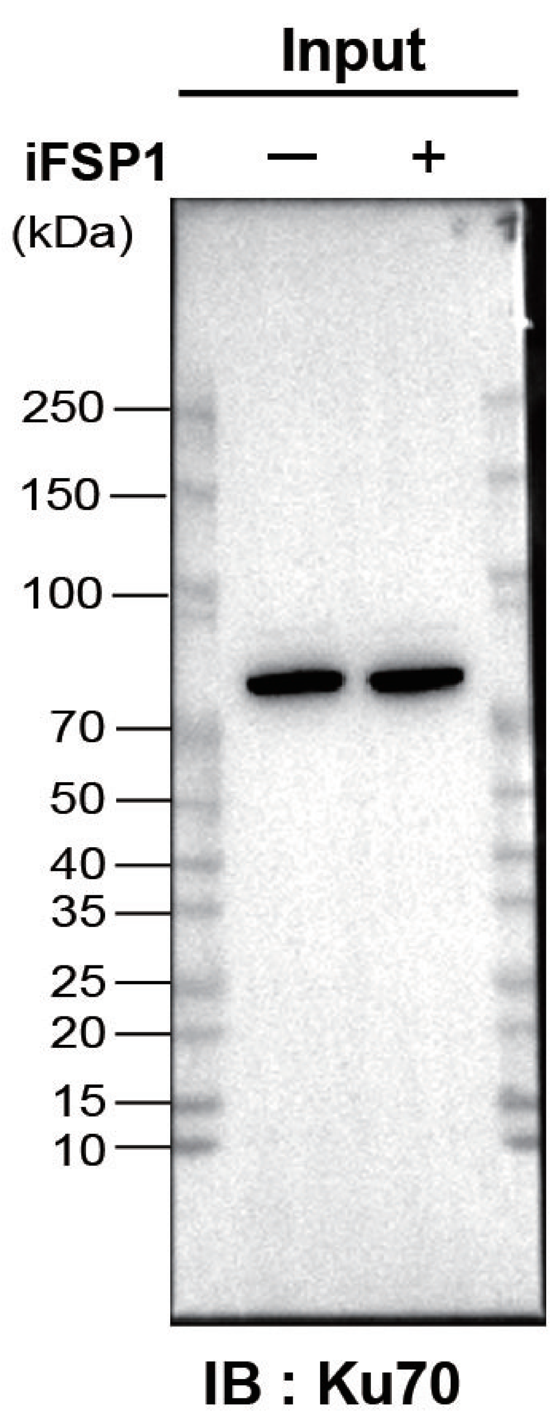

Figure S6D

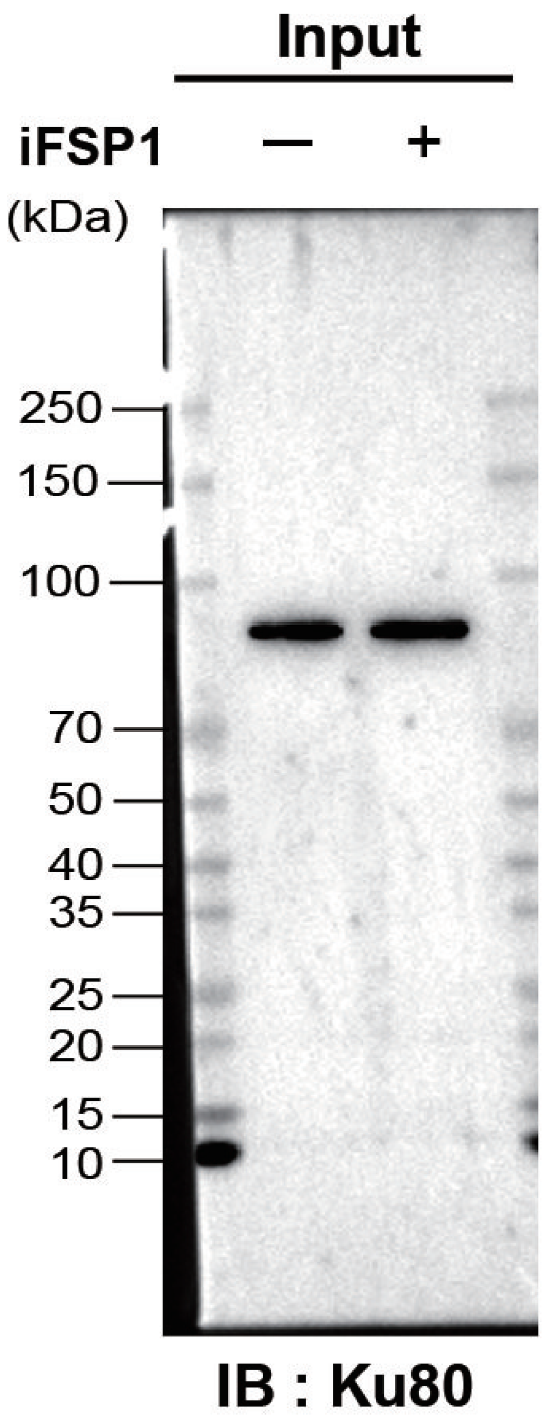

Figure S6F

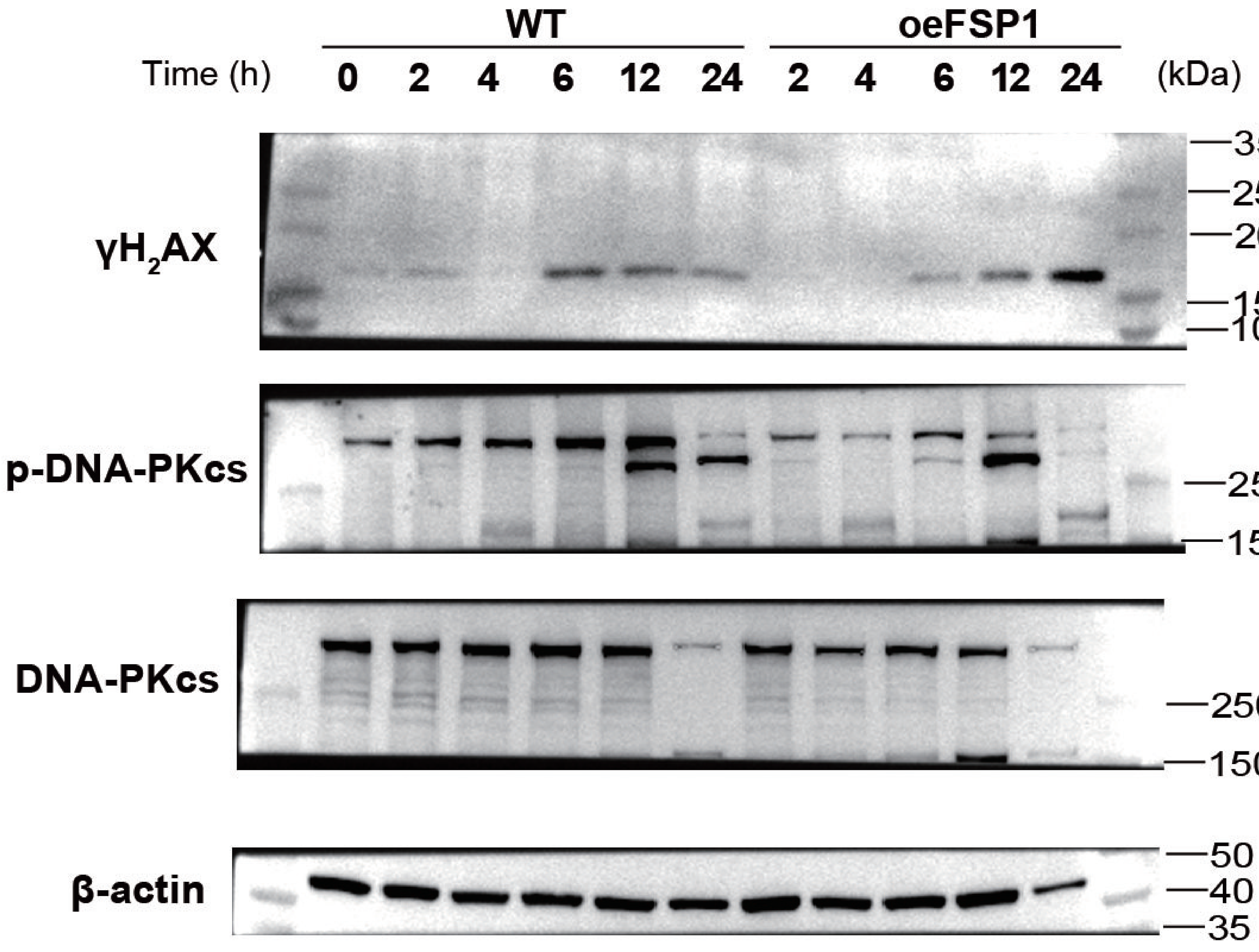

Supplement: Supplementary file 1 — original data files [file 41418_2024_1263_MOESM1_ESM.pdf]
