# Supplementary material for: FSP1 inhibition enhances olaparib sensitivity in BRCA-proficient ovarian cancer patients via a nonferroptosis mechanism
Source: Cell Death Differ. 2024 Feb 19;31(4):497–510. doi: 10.1038/s41418-024-01263-z (PMC11043371; doi:10.1038/s41418-024-01263-z)
Supplement: Supplementary file 2 — Supplementary_information [file 41418_2024_1263_MOESM2_ESM.docx]

**Supplementary files list**

**
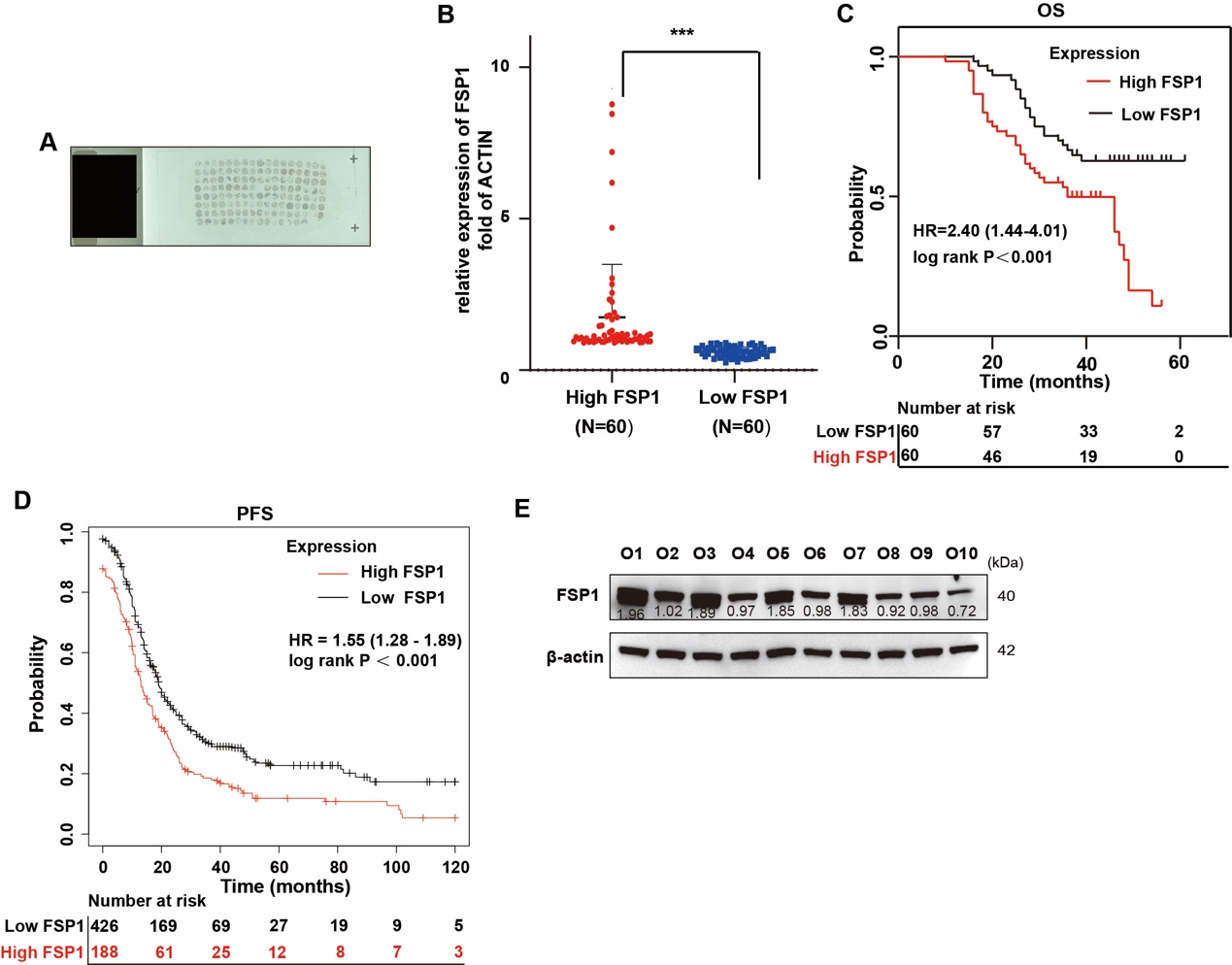
**

**Figure S1. FSP1 overexpression is a risk predictor for poor prognosis in OC. A,** Immunohistochemistry analysis of FSP1 expression in ovary tumors (N=131) and normal ovary (N=3). **B,** qPCR determined FSP1 expression in clinical samples from OC patients(N=120). Patients were grouped into FSP1-high (N=60) or FSP1-low group (N=60) depending on the median expression of FSP1. **C,** Survival analysis of overall survival (OS) was performed in the FSP1 high/low group (**B**). **D,** FSP1 overexpression was negatively related to the progression-free survival (PFS) in OC in the public database. **E,** Immunoblot analysis of FSP1 in the 10 primary OC tumor specimens of PDOs.


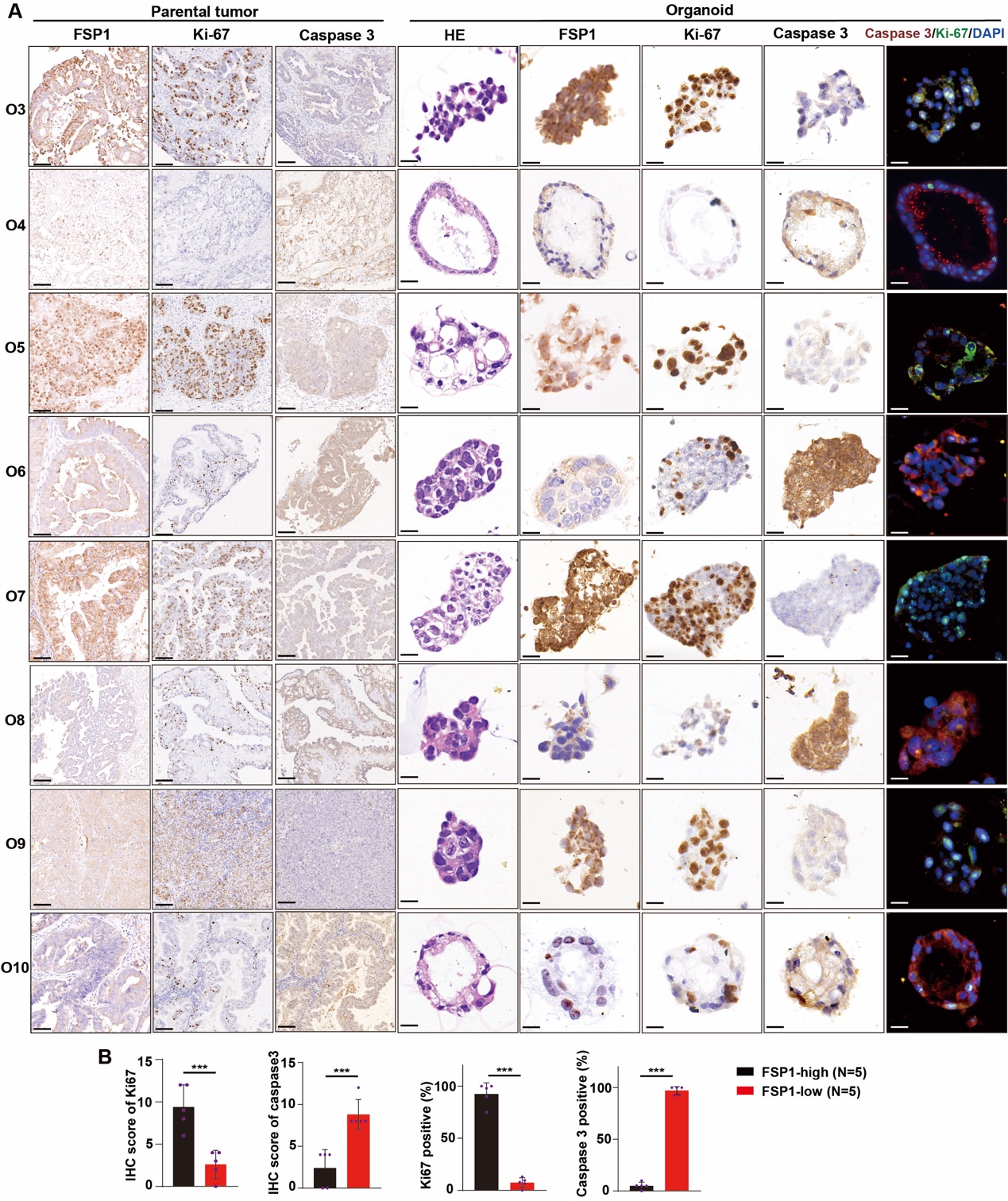


**Figure S2.** **FSP1 high level is associated with high cell proliferation. A,** Representative images of H&E, IHC (FSP1, Ki-67 and Caspase 3) and IF staining (Ki-67 and Caspase 3) in OC samples and organoids (N=10). **B,** Quantification of FSP1, Ki-67 and Caspase 3 protein expression in the FSP1-high (N=5) and FSP1-low (N=5) OC and PDOs.


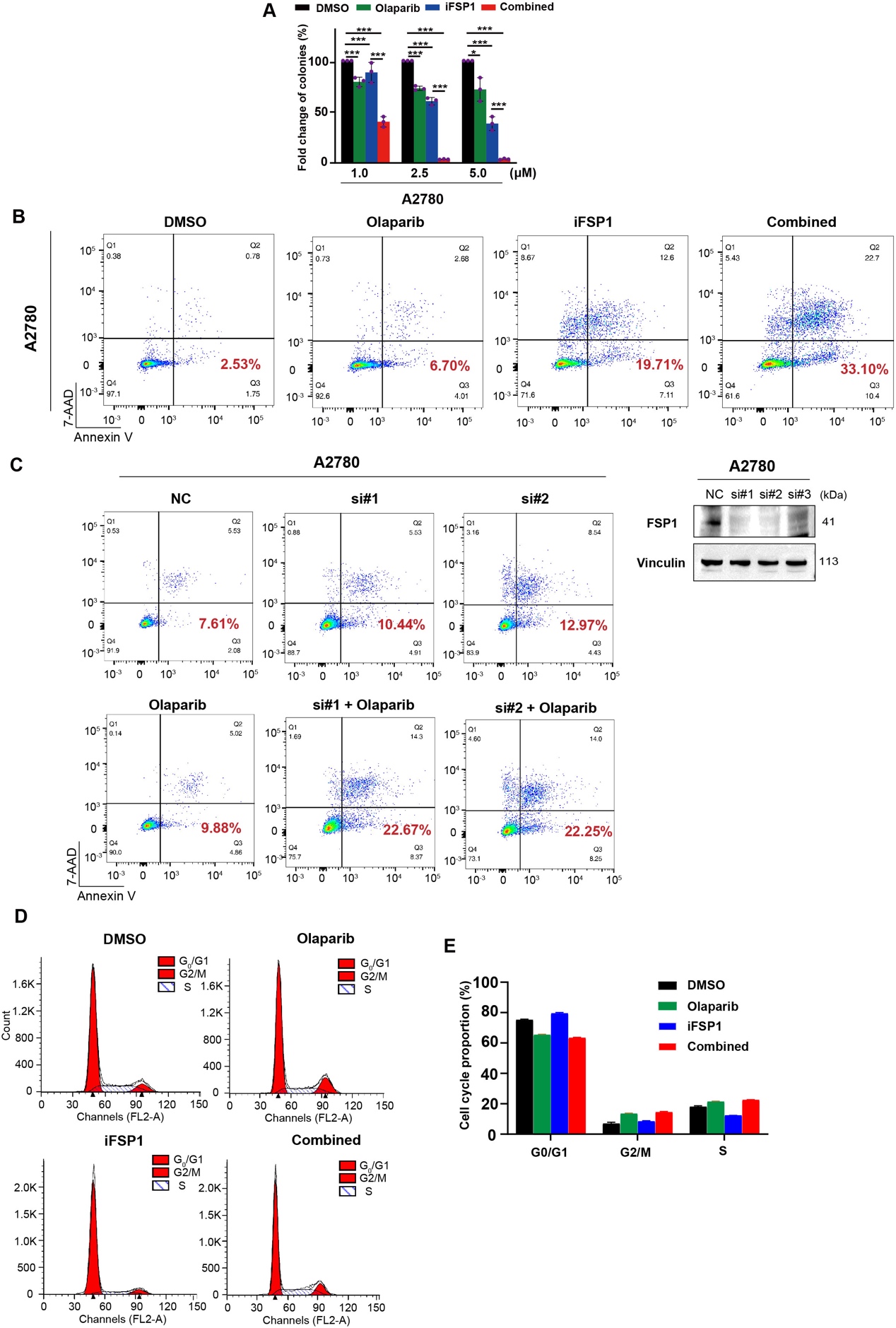


**Figure S3. The synergistic lethality of FSP1 inhibition and Olaparib in BRCA-proficient OC. A,** The number and area of colonies was calculated in the A2780 cells after olaparib, iFSP1 and in combination treatment. ****P*＜0.001. **B,** Images of flow cytometry analysis of Annexin V and 7-AAD markers in the HO-8910 cells treated with olaparib (10 μM) and iFSP1 (10 μM) singly and in combination for 24 hours. **C,** Flow cytometry assessed the apoptotic cells in A2780 cells in response to olaparib (10 μM) after silencing FSP1. **D,E** The cell cycle distribution analysis in the HO-8910 cells treated with olaparib (10 μM) and iFSP1 (10 μM) singly and in combination for 24 hours.


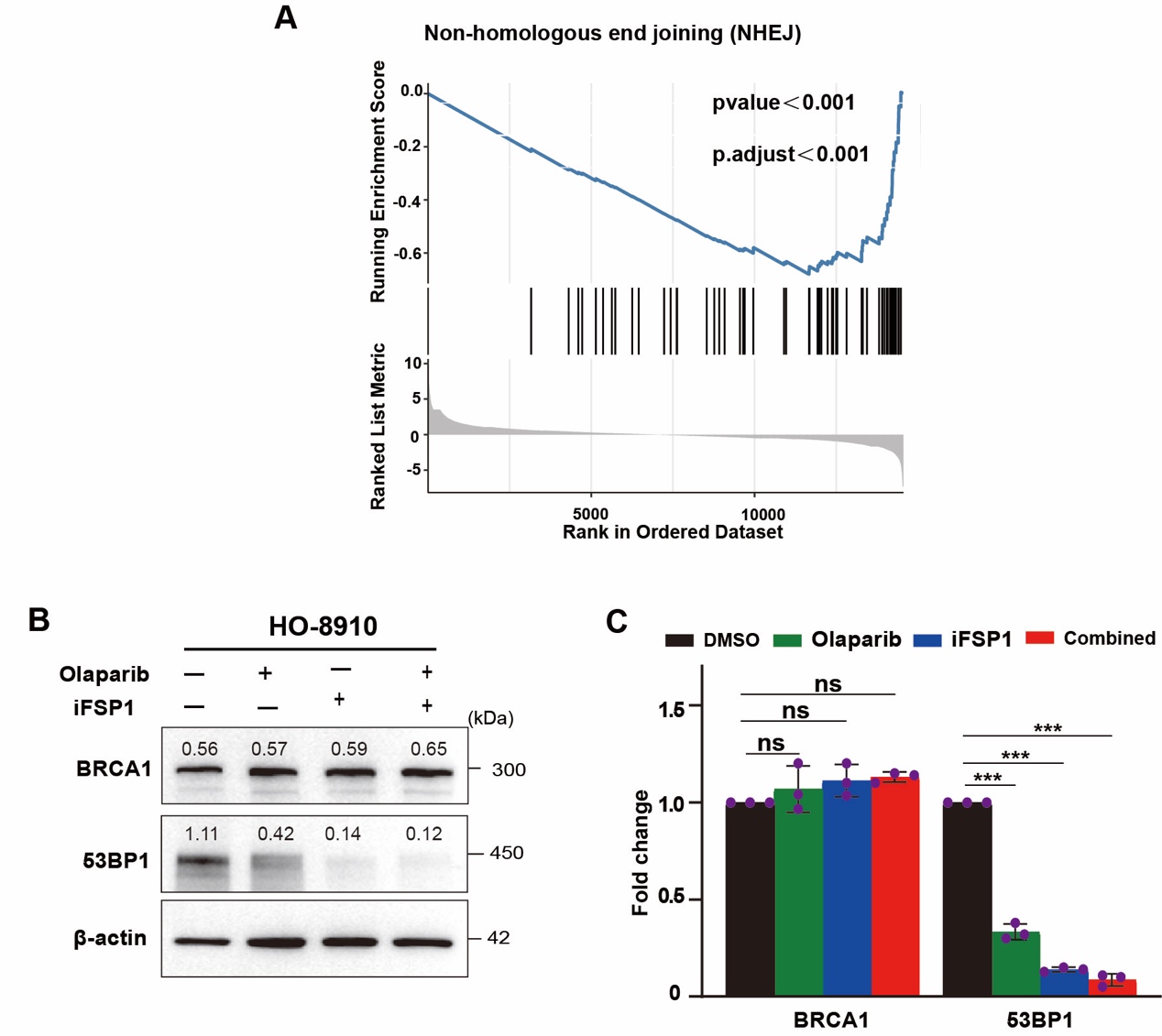


**Figure S4.** **The combination of agents impairs NHEJ activity in BRCA-proficient OC. A,** The GSEA enrichment of non-homologous end joining (NHEJ) pathway was performed using the DEGs between the DMSO and combined treatment in PDO O1 (N=3 per group). **B**,**C**, Immunoblot analysis of 53BP1 and BRCA1 levels in each group in HO-8910 cells. The relative expression of 53BP1 and BRCA1 to β-actin has been labeled (**B**), and the fold change values have been quantified (**C**). ****P*＜0.001; ns, no significance.


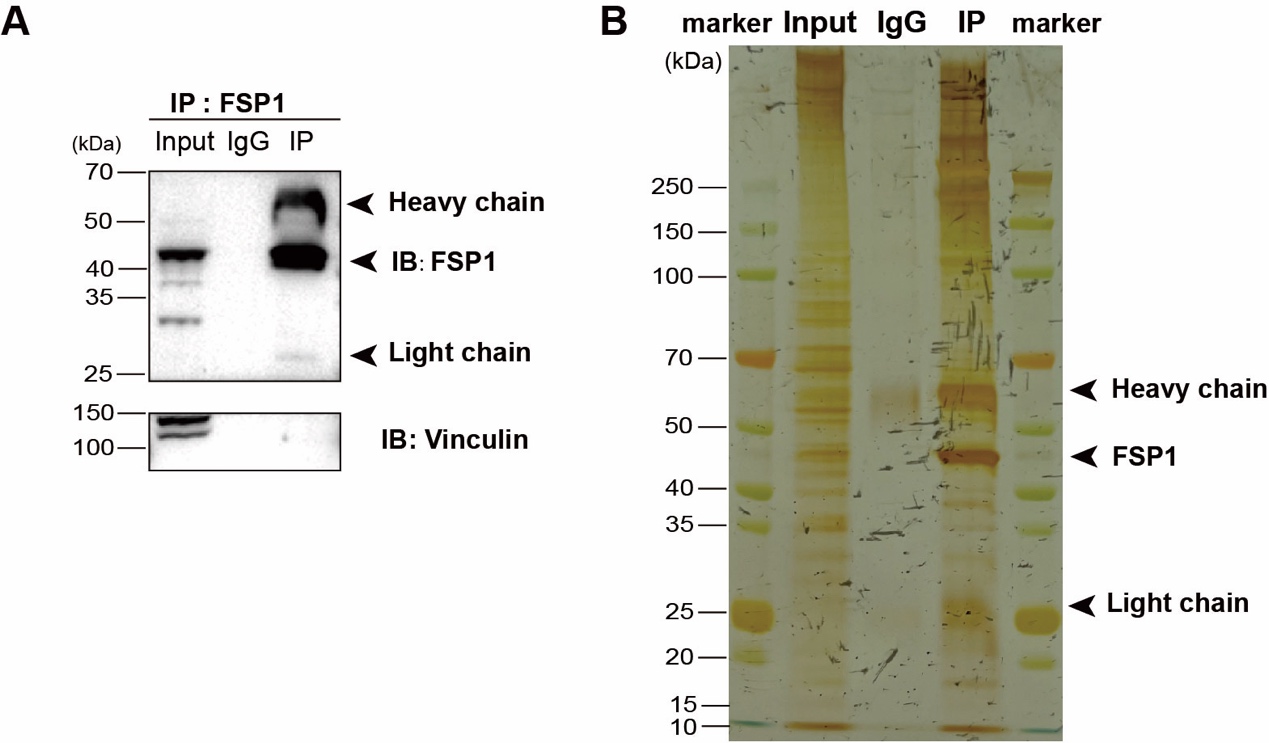


**Figure S5. The quality control of IP with anti-FSP1 antibody. A,** Representative images of immunoblot analysis of IP with anti-FSP1 antibody. **B,** Representative images of silver staining of IP with anti-FSP1 antibody.


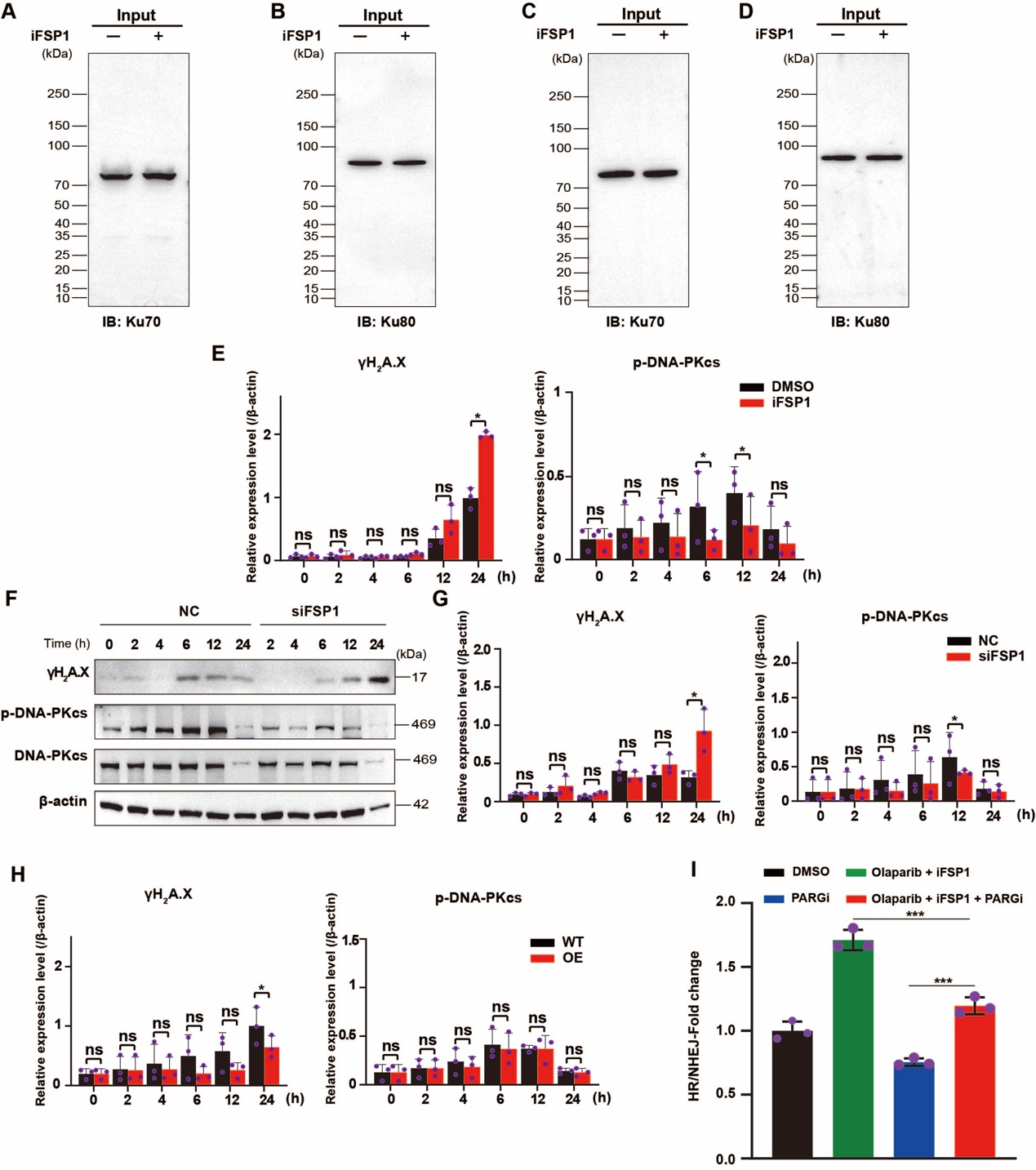


**Figure S6. FSP1 regulates NHEJ activity through PARylation of Ku70. A,B,C,D** Representative images of immunoblot analysis of input with anti-Ku70 antibody (**A,C**) or anti-Ku80 antibody (**B,D**) in HEK293t cells. **E**, Relative expression of γH2A.X and phosphate-DNA-PKcs to β-actin was compared between the DMSO and iFSP1 treatment in HO-8910 cells. **F,G,** Immunoblot analysis determined the levels of γH2A.X, DNA-PKcs and phosphate-DNA-PKcs (p-DNA-PKcs) in the HO-8910 cells after silencing FSP1 by siRNAs. **H,** Relative expression of γH2A.X and p-DNA-PKcs was compared between the wild type (WT) and FSP1-overexpressed (OE) HO-8910 cells. **I,** Fold change of HR/NHEJ ratio in each group was shown after rescued by the PARG inhibitor PDD00017273 (PARGi) in the HO-8910 cells.


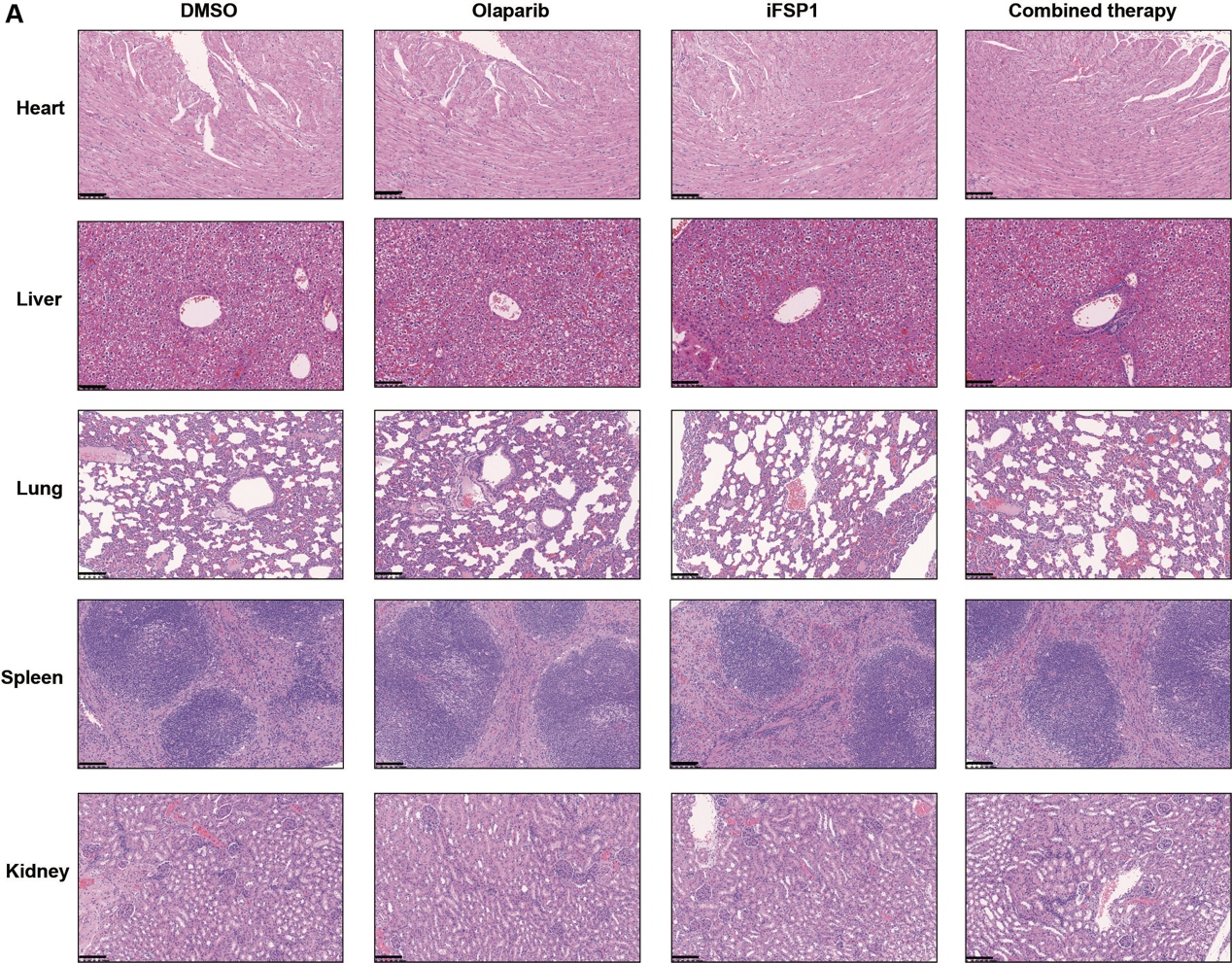


**Figure S7. Safety of the combination treatment in xenograft mouse model. A,** Representative images of H&E staining of sections from heart, liver, lung, spleen and kidney in each group.

**Supplementary Table Legends**

**Table S1**. **Patients’ characteristics.**

**Table S2. Primary antibodies used in the study.**

**Table S3. Sequences of Primers and siRNAs.**
